# Chemically Gradient Ordered Nanodomains Enable Large Tensile Ductility in Gigapascal Lightweight Refractory High‐Entropy Alloys

**DOI:** 10.1002/advs.76407

**Published:** 2026-07-06

**Authors:** Wei Zhang, Dingshun Yan, Yong Zhang, Zhiming Li

**Affiliations:** ^1^ School of Materials Science and Engineering Central South University Changsha China; ^2^ State Key Laboratory of Powder Metallurgy Central South University Changsha China

**Keywords:** chemical stability, composite material, ductility, elongation, hypersonic speed, materials science, refractory, thermal stability, ultimate tensile strength

## Abstract

High‐strength lightweight refractory alloys are vital components serving in harsh environments with high mechanical loads at elevated temperatures, e.g., in aero‐engines and hypersonic vehicles. Despite the long‐term efforts on strengthening these materials, they usually show low ductility, particularly at room temperature, severely limiting their practical applications. Here, we introduce a concept to design extraordinarily ductile high‐strength lightweight refractory high‐entropy alloys (RHEAs) by introducing dispersed chemically gradient ordered nanodomains (CGONs, 1–3 nm) coherent with the disordered matrix. Chemical composition of the shell in the CGON is similar to that of the adjacent disordered matrix, and it varies gradually from the shell to the core. Such architecture not only enhances the thermodynamic stability of the CGONs but also facilitates stress transfer across the interfaces by reducing interfacial strain energy. The concept is realized in an Nb‐Zr‐Ti‐Ta‐Al RHEA system showing low mass density of 6.48 g/cm^3^, large uniform tensile elongation of 21.5%, elongation‐to‐failure of 47.8%, and high yield strength exceeding 1.0 GPa at room temperature. Further, the enhanced thermal stability of the CGONs contributes to the excellent high‐temperature performance of the lightweight RHEA. The provided insights are thus important in guiding the development of ductile and ultra‐strong lightweight refractory materials for key engineering applications.

## Graphical abstract

A high‐strength lightweight refractory high‐entropy alloy achieves extraordinary ductility owing to the introduction of coherent chemically gradient ordered nanodomains (CGONs). The CGONs not only serve as effective barriers to dislocation motion (strengthening) but also significantly promote dislocation nucleation and multiplication (ductilizing) upon loading at critically high stress levels.

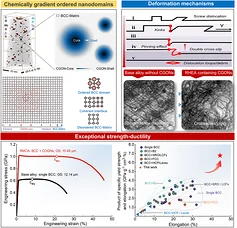

## 1 Introduction

Refractory alloys have been highly demanding for high‐temperature structural applications, e.g., in aero‐engines and power‐generation sectors, where materials must withstand extreme thermal and mechanical loadings [1, 2, 3]. Conventional Ni‐based superalloys have served in the associated industries, but they have gradually reached their service temperature limits, and the relatively high cost and high mass density also lead to constraints in applications [4, 5]. Therefore, new refractory alloys have long been pursued to achieve optimized mechanical properties at varying temperatures (e.g., 873–1273 K) with reduced mass density and cost [6]. In recent years, the emergence of refractory high‐entropy alloys (RHEAs) has introduced a high flexibility for designing refractory materials with improved performance in a practically infinite compositional space. The RHEAs usually contain high concentrations of Group IV, V, and VI elements, e.g., W, Mo, Hf, Nb, and Ta, which offer exceptional mechanical strength within a wide temperature range [7]. However, apart from high mass densities, these heavy elements also result in low ductility, particularly at room temperature, limiting their processing abilities and hindering them from practical light‐weight structural applications [8, 9, 10].

The low ductility of previous RHEAs at room temperature is primarily attributed to the insufficient dislocation multiplication and interactions during plastic deformation [11, 12]. Efforts have been devoted to improving the strength‐ductility synergy of RHEAs, e.g., by interstitial alloying [13, 14] and grain refinement [15]. However, these approaches often fail to enhance the number density of active dislocation nucleation sites, or cannot optimize the uniformity of dislocation slips, and hence, the tensile ductility can hardly be improved [16]. Also, conventional precipitation has been applied in RHEAs, which can exhibit a significant strengthening effect, yet at poor tensile ductility. For instance, ordered B2 precipitates showing sharp interfaces and significantly different chemical compositions with the disordered body‐centered cubic (BCC) matrix can enable high yield strength in RHEAs. However, previous BCC‐B2 dual‐phase RHEAs also suffer from poor tensile ductility due to the large differences in chemical compositions and corresponding mismatch of lattice parameters between the B2 phase and the BCC matrix, which are prone to induce significant stress concentrations at the BCC‐B2 interfaces during stress loading [17, 18, 19].

To address the above problems, we propose a concept to design extraordinarily ductile high‐strength lightweight RHEAs by introducing dispersed chemically gradient ordered nanodomains (CGONs). Unlike conventional B2 precipitates in a disordered BCC matrix, the CGONs are nanoscale coherent regions (1–3 nm) with spatially gradient chemical compositions from the shell to the core, and the chemical composition of the shell in the CGON is similar to that of the coherent disordered BCC matrix. This unique structural and chemical design brings about distinct benefits: (i) enhances thermodynamic stability of the nanostructures by reducing interfacial strain energy; (ii) facilitates efficient stress transfer across interfaces so that stress concentrations at interfaces can be relieved; (iii) provides effective barriers to dislocation motion, delivering remarkable strengthening effect; (iv) stimulates dislocation nucleation and multiplication by the atomic‐scale stress fields in CGONs upon deformation, leading to ductilizing effect; (v) promotes the dispersions of nano‐scale strain fields and prevents the localized accumulation of dislocations to homogenize the plastic flow, thereby optimizing the strength‐ductility synergy in the RHEAs.

In this work, we realize the above concept by developing a non‐equiatomic lightweight RHEA with nominal composition Nb_32_Zr_29.5_Ti_28_Ta_2.5_Al_8_ (at. %) based on systematic thermodynamic calculations (See Methods and Figure S1). The alloy design rationale integrates several key aspects: (1) a BCC structured massive solid solution matrix consisting of multiple principal elements; (2) significant thermodynamic driving force, e.g., large negative mixing enthalpy within the supersaturated solid solution that trigger the formation of stable ordered nanodomains; (3) a gradual transition of chemical composition from shell to core within the nanostructured domains, i.e., to form CGONs with the local chemical composition of the shell similar to that of the adjacent disordered BCC matrix. Accordingly, the principal elements Nb, Ti, and Zr are employed based on the Nb‐Ti‐Zr ternary phase diagram (Figure S1d) to construct the stable BCC structure with high lattice distortion and enhanced friction stress against dislocation motion [8, 20]. Ta and Al are selected to promote the formation of CGONs, since the negative mixing enthalpies among the alloying element pairs can provide thermodynamic possibilities for designing the targeted nanostructures. The RHEA was synthesized by arc‐melting, cold‐rolling, and annealing (1123 K for 5 min). Processing details are provided in Methods. To clarify the mechanisms responsible for the enhanced mechanical performance of the lightweight RHEA with CGONs, a reference Nb_34.0_Ti_33.0_Zr_33.0_ (at. %) ternary base alloy (BA) without CGONs was also prepared and investigated.

## 2 Results

### 2.1 Micro‑, Nano‑ and Atomic‑Scale Structures

Figure 1 shows the micro‐, nano‐, and atomic‐scale structures of the present lightweight RHEA after annealing at 1123 K for 5 min. A body‐centered cubic (BCC) matrix is identified based on the X‐ray diffraction (XRD) pattern (Figure 1a) and electron backscatter diffraction (EBSD) phase map (Figure 1b). The average grain size of the RHEA was measured to be 10.32 ± 3.64 µm (Figure S2a). The multiple elements are homogeneously distributed at the micro‐scale as revealed by back‐scattered electron (BSE) imaging and corresponding energy‐dispersive X‐ray spectrometry (EDS) analysis (Figure S3a).

**FIGURE 1:**
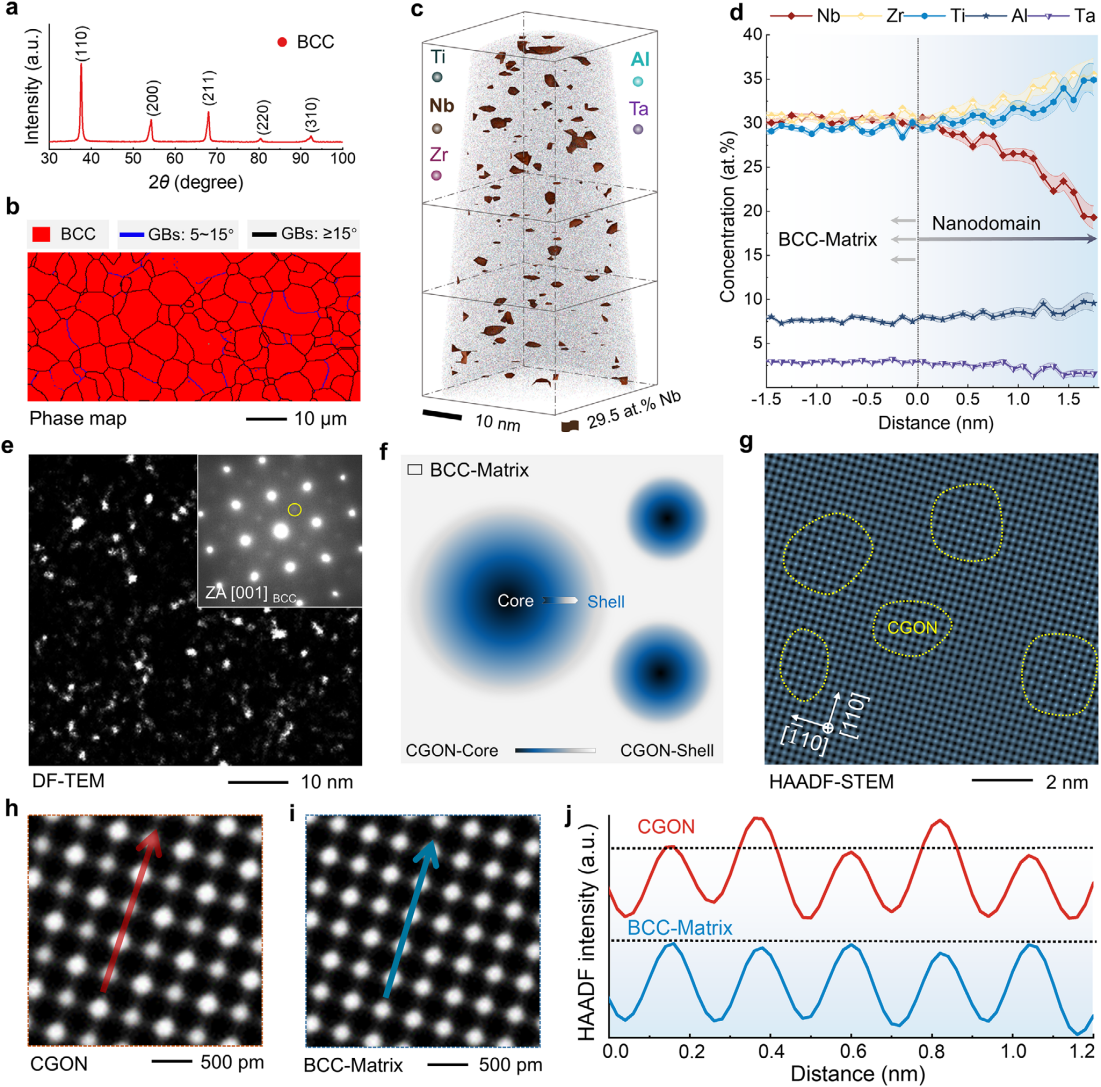
Structure of the lightweight RHEA. (a, b) XRD pattern and EBSD phase map showing a BCC matrix. “a.u.” refers to “arbitrary unit”. The fundamental BCC peaks show a subtle asymmetry indicative of the existence of coherent nanoscale domains with very small lattice mismatch. (c) 3D reconstruction map of a typical APT tip with iso‐composition surface of 29.5 at. % Nb showing nanodomains in the matrix. (d) 1D‐proxigram analysis showing the concentrations of various elements with respect to distance, revealing the chemical gradients in the nanodomains. (e) DF‐TEM image taken with the (001) superlattice spot marked by the yellow circle in the inset. (f) Schematic of the chemically gradient ordered nanodomains (CGONs) randomly distributed in the disordered BCC matrix. (g) HAADF‐STEM image taken under [001]_BCC_ zone axis showing the atomic‐scale structure of the CGONs. (h, i) Arrangements of atomic columns in the CGON and BCC matrix, respectively. (j) Typical atomic intensity profiles with respect to distance along the [110] directions marked by the arrows in (h, i), respectively.

According to the atom probe tomography (APT) reconstruction map shown in Figure 1c, nanosized domains are present in the otherwise homogeneous matrix, as outlined by the iso‐composition surface of 29.5 at. % Nb in the 3D map. The average diameter and volume fraction of the nanodomains are 2.86 ± 1.28 nm and 9.6 ± 0.35%, respectively, based on multiple APT datasets (Figure S5). The 1D‐proxigram analysis in Figure 1d reveals that the inner regions of the nanodomains are enriched with Zr, Ti, and Al, while depleted with Nb and Ta. More importantly, the nanodomains distinctly show gradient chemical compositions, i.e., Zr, Ti, and Al concentrations are gradually increasing from the shell to the core regions, while Nb and Ta exhibit the opposite trend. The core region with a radius of ∼0.5 nm holds an average composition of Nb_23.4_Ti_33.8_Zr_33.1_Ta_2.1_Al_8.8_ (at. %), whereas the compositions of the shell region are similar to that of the adjacent BCC matrix, i.e., Nb_29.7_Ti_32.8_Zr_30.5_Ta_2.8_Al_7.5_ (at. %). Further, the selected area electron diffraction (SAED) pattern and corresponding dark‐field (DF) transmission electron microscopy (TEM) image in Figure 1e demonstrate the coherent ordered B2 structure of the nanodomains. Figure 1f schematically illustrates the ordered nanodomains with chemical gradients, i.e., CGONs described above, randomly distributed in the disordered BCC matrix.

The structure of the CGONs is further characterized at the atomic scale by high‐angle annular dark field (HAADF) scanning TEM (STEM) analysis. The atomic‐resolution HAADF‐STEM images in Figure 1g and Figure S3b confirm the coherent interfaces between the ordered CGONs and the disordered BCC matrix. Figure 1h,i displays the arrangements of atomic columns in the CGON and BCC matrix, respectively. Accordingly, the typical atomic intensity profiles with respect to distance along the [110] directions marked by the arrows in Figure 1h,i are constructed, as shown in Figure 1j. The intensities of atomic columns in the BCC matrix are identical, whereas the atomic columns in the CGON show periodic fluctuation of intensities. This further validates the ordered structure of the CGONs and the disordered structure of the BCC matrix.

For comparison, the multi‐scale structures of the reference Nb_34.0_Ti_33.0_Zr_33.0_ (at. %) ternary base alloy under identical processing conditions have also been investigated. The base alloy exhibits a BCC structure with an average grain size of 11.89 ± 2.26 µm (Figure S4a,b and S2b). The three principal elements, i.e., Nb, Ti, and Zr, are uniformly distributed in micro‐scale as revealed by EDS analysis in Figure S4c. The bright‐field STEM image (Figure S4d) and HAADF‐STEM image (Figure S4f) taken along the [001] zone axis demonstrate a single disordered BCC structure. Further, the SAED pattern (Figure S4e) and the fast Fourier transform (FFT) pattern (inset in Figure S4f) show that only diffraction spots of the disordered BCC structure are reflected, confirming the absence of CGONs and secondary phases in the base alloy.

Atomic‐scale chemical characteristics of the CGONs in the RHEA are further probed by spherical aberration‐corrected STEM analysis. Prior to the demonstration of the atomic‐scale chemistry, the inverse fast Fourier transformation (IFFT) image (Figure 2a) and the corresponding FFT patterns (Figure 2b,c) are used to locate the CGON and BCC matrix by yellow and red rectangles in Figure 2a, respectively. Figure 2d shows the atomic‐resolution STEM‐EDS maps of Nb, Zr, Ti, Ta, and Al obtained along the [001] zone axis. Slight compositional fluctuations of Zr, Ti, and Al, opposite to that of Nb and Ta, can be observed in the CGON, which is qualitatively consistent with the chemical features identified by APT analysis (Figure 1d). Yet, quantitative information on the chemical compositions of CGON can hardly be obtained by the STEM‐EDS analysis because the size of the CGONs (2.86 ± 1.28 nm) is significantly smaller than the excitation volume of characteristic X‐rays, causing the acquired signal to be an inseparable mixture of contributions from the CGONs and the surrounding matrix.

**FIGURE 2:**
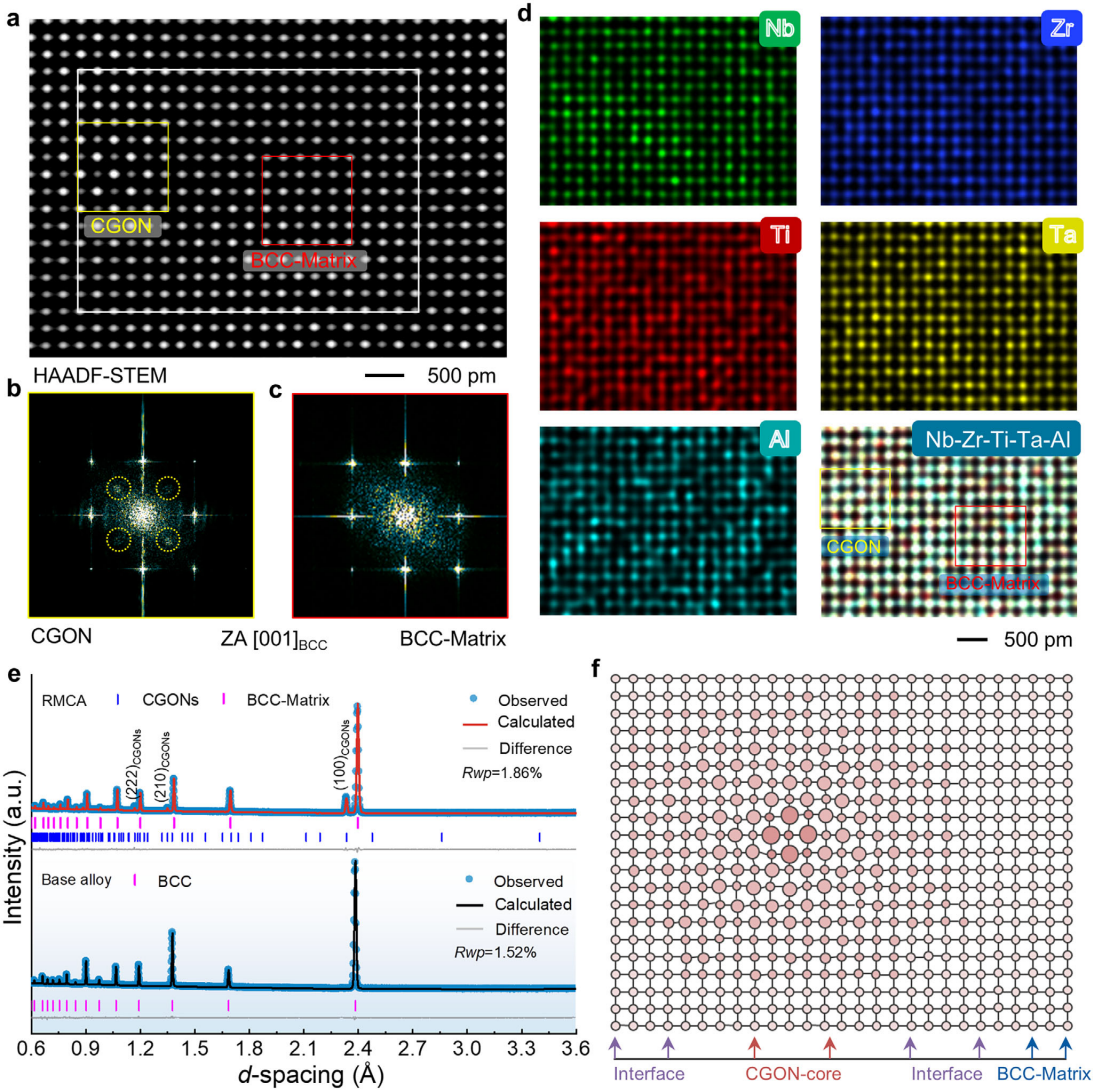
Atomic‐resolution chemical characteristics and high‐resolution neutron diffraction analysis. (a) Inverse fast Fourier transformation (IFFT) image corresponding to the HAADF‐STEM image in Figure S3, showing the atomic‐scale structure of the CGON and the BCC‐matrix. (b, c) Fast Fourier transform (FFT) patterns of the CGON and the BCC‐matrix regions are marked in (A), respectively. (d) Atomic‐scale STEM‐EDS maps of the RHEA for individual elements Nb, Zr, Ti, Ta, and Al taken with the [001]_BCC_ zone axis and the close‐up map with combined signals of Nb, Zr, Ti, Ta, and Al. (e) Refined neutron diffraction patterns of the RHEA and the base alloy. (f) Schematic of the gradient change of local lattice size in the CGON from the core toward the shell.

To further reveal the details of lattice structures in the lightweight RHEA and the base alloy, high‐resolution neutron diffraction analysis was performed on the bulk samples. Figure 2e shows the neutron diffraction patterns of the RHEA (red line) and the base alloy (black line) after the Rietveld refinement. The base alloy exhibits sharp diffraction peaks, which can be indexed as a single BCC phase, with a lattice parameter of 3.367 Å. For the RHEA, besides the diffraction peaks of the disordered BCC matrix, the (100), (210), and (222) superlattice reflections emergence at *d*‐spacings of 2.329, 1.345, and 1.167 Å, respectively, providing macroscopic direct evidence for the existence of CGONs. The average lattice parameters of the BCC matrix and the CGONs are 3.383 and 3.293 Å, respectively, suggesting a mean apparent lattice misfit of 2.63%. Note that this mean apparent lattice misfit value cannot reflect the misfit at the exact interface between the CGONs and the disordered BCC matrix due to the gradual change of local lattice size in the CGONs by the distinct chemical gradient. As schematically illustrated in Figure 2f, the lattice expansion in the CGON is gradually accommodated and reduced by the chemical gradient from the core toward the shell. This results in minimal lattice misfit at the exact coherent interface between CGON and disordered BCC matrix, which is estimated to be ∼0.018% by Vegard's law [21] using the chemical compositions of the disordered BCC matrix and the shells in the CGONs from the above APT analysis.

### 2.2 Tensile Behavior

Figure 3a shows the representative tensile engineering stress‐strain curves of the RHEA and the base alloy (both annealed at 1123 K for 5 min) at room temperature. The tensile yield strength of the RHEA (∼1032 MPa) is increased by ∼67.3% compared with that of the base alloy (∼617 MPa). Moreover, the RHEA shows superior uniform elongation reaching ∼21.5%, nearly tripling that of ∼8.2% for the base alloy. The elongation‐to‐failure (∼47.8%), which is ∼75% larger than that of the base alloy (∼27.3%). Figure 3b exhibits the profiles of true stress and strain hardening rate (SHR) with respect to true strain. The SHR of the base alloy quickly drops with increasing true strain during the entire plastic deformation process, resulting in a low SHR and a relatively small uniform elongation. Interestingly, the present RHEA exhibits a continuously increasing SHR over a wide true strain range (∼3.0%–14.5%) after the drop of SHR in the initial plastic deformation stage, which corresponds to significantly higher SHR and a large uniform elongation of 21.5%. This enhanced uniform elongation of the RHEA is further demonstrated by the 2D strain distribution maps at different loading stages measured by the digital image correlation (DIC) method (Figure S6a), revealing homogeneous macroscopic plastic deformation at engineering strains from ∼2% to ∼25%. Following the onset of necking, the local strain increases dramatically to ∼180% before fracture (Figure S6b).

**FIGURE 3:**
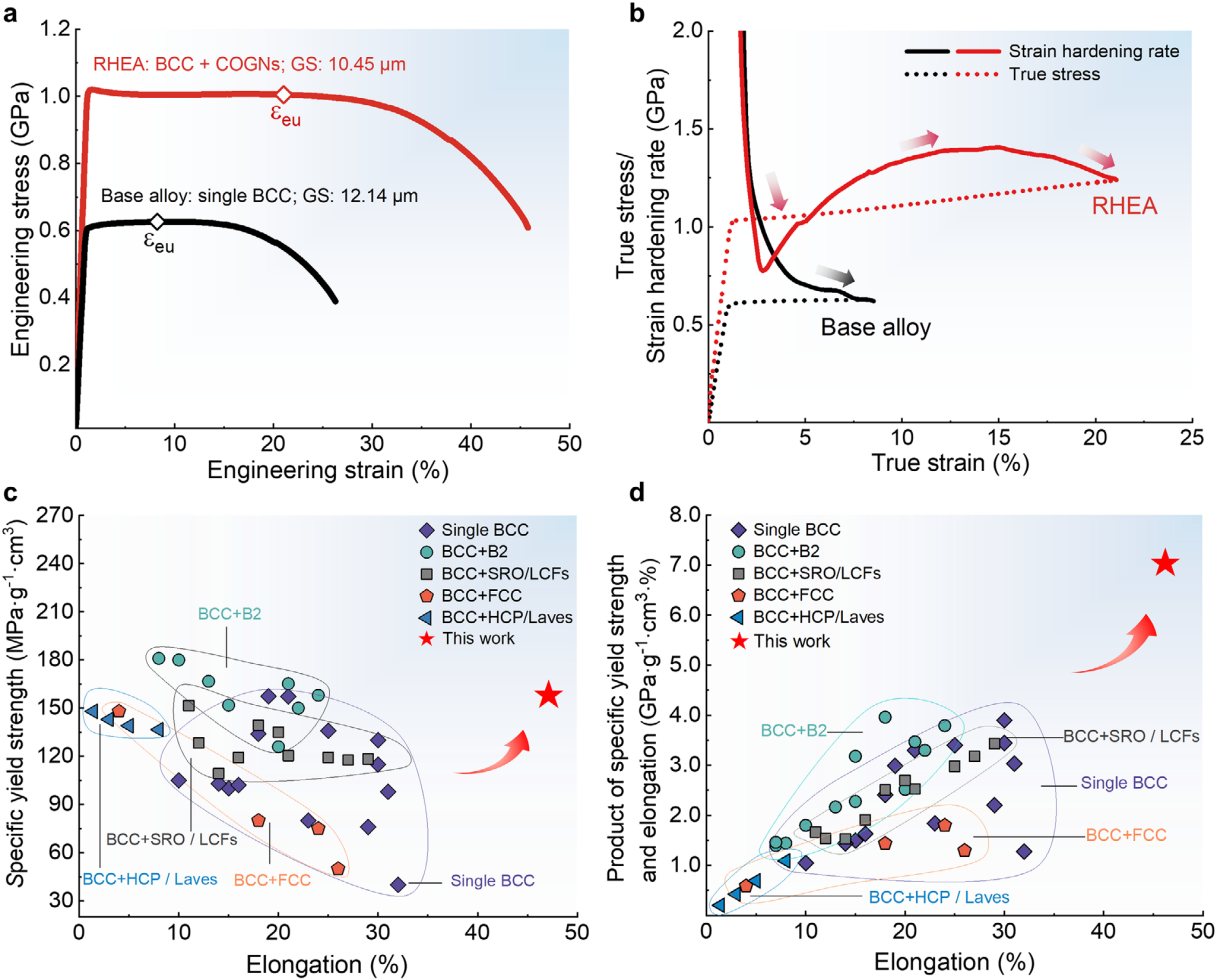
Mechanical properties of the lightweight RHEA. (a) Typical engineering stress‐strain curve at ambient temperature for the RHEA (red curve) and the base alloy (black curve). (b) Strain hardening rate and true stress with respect to true strain for the RHEA and the base alloy; (c) Plot of tensile elongation with respect to specific yield strength for the present RHEA and the other refractory alloy systems including single‐phase solid solution alloys [22, 23], single‐phase BCC alloys containing short‐range order (SRO) or local chemical fluctuations (LCFs) [24, 25, 26], BCC+B2 dual‐phase alloys [27, 28, 29], and BCC‐based alloys reinforced with face‐centered cubic (FCC) or hexagonal close‐packed (HCP) Laves phases [30, 31, 32, 33, 34, 35]. (d) Plot of the products of specific yield strength and tensile elongation with respect to tensile elongation for the various alloy systems.

Compared to the previous BCC refractory alloys reinforced with conventional B2 precipitates, the present CGON‐strengthened RHEA demonstrates a superior combination of ductility and strength at room temperature, as shown in Figure S7. Further, Figure 3c,d highlights the excellent synergy of low mass density, extraordinary ductility, and high strength. The specific yield strength, i.e., the ratio of yield strength to mass density of the RHEA approaches 159.3 MPa·g^−1^·cm^3^, exceeding those of other reported refractory alloy systems including single‐phase solid solution alloys [22, 23], single‐phase BCC alloys containing short‐range order (SRO) or local chemical fluctuations (LCFs) [24, 25, 26], BCC+B2 dual‐phase alloys [27, 28, 29], and BCC‐based alloys reinforced with face‐centered cubic (FCC) or hexagonal close‐packed (HCP) Laves phases [30, 31, 32, 33, 34, 35]. More notably, the product of specific yield strength and tensile elongation of the RHEA reaches 7.6 GPa·g^−1^·cm^3^·%, which is unprecedentedly high compared to those obtained in previous RHEAs, as shown in Figure 3d.

Besides the unprecedented room‐temperature properties, the present RHEA also possesses considerable mechanical performance at elevated temperatures up to 1273 K, demonstrating its intrinsic refractory nature. As shown in Figure S8a, the RHEA exhibits substantial strain hardening behavior at 773 K, and a notable softening phenomenon upon tensile loading only occurs when the temperature reaches 1073 K and above. Further, the low mass density of the present RHEA endows it with superior specific tensile yield strength at a wide temperature range from room temperature to 1273 K, compared to the previous superalloys and emerging multi‐principal‐element alloys (Figure S8b).

### 2.3 Structural Evolution Upon Tensile Deformation

The evolution of microscale deformation structures at local strains (*ε*_loc_) of ∼8%, ∼38%, and ∼85% are shown by EBSD kernel average misorientation (KAM) and inverse pole Figure (IPF) maps in Figure 4a–c and Figure S9, respectively. The deformation microstructure analysis confirms that dislocations prevail under tensile straining, whereas deformation twinning and phase transformation are absent. In Figure 4a, KAM maps reflecting the distribution of geometrically necessary dislocations (GNDs) suggest that the dislocations are concentrated primarily near grain boundaries at the early uniform deformation stage (*ε*_loc_ ∼8%). With the proceeding of plastic deformation, dislocations are distributed throughout the entire grains at the later uniform deformation stage (*ε*_loc_ ∼38%, Figure 4b) and the post‐necking stage (*ε*_loc_ ∼85%, Figure 4c). Quantitative analysis suggests that the dislocation density significantly increases from 3.89 × 10^13^ m^−2^ at *ε*_loc_ of ∼8% to 1.37 × 10^15^ m^−2^ at *ε*_loc_ of ∼85%.

**FIGURE 4:**
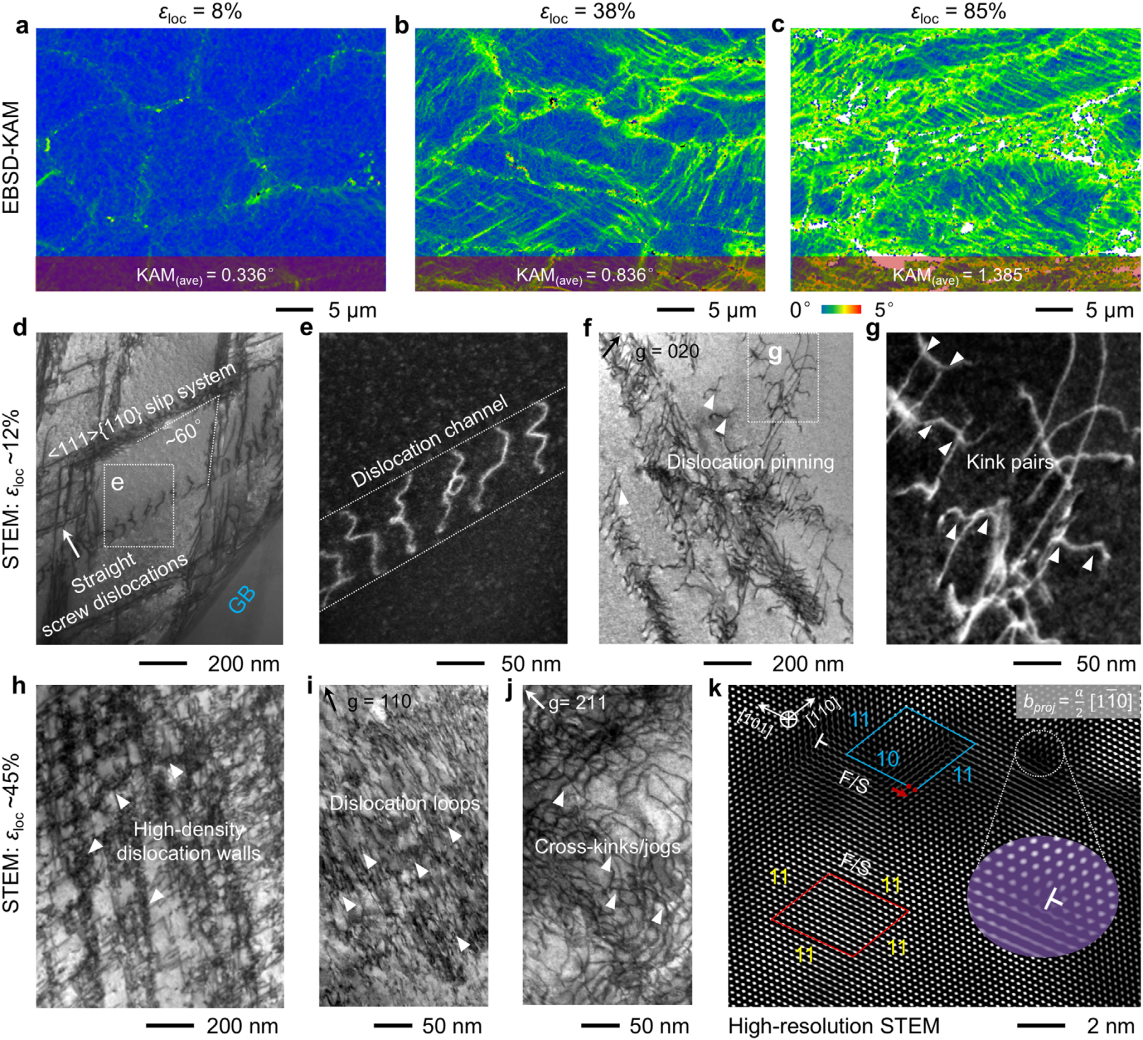
Deformation substructures of the lightweight RHEA. (a–c) EBSD KAM maps showing deformation‐induced local misorientation features at different local strain levels (∼8%, ∼38%, and ∼85%). (d) BF‐STEM image showing the deformation substructures at a local strain of ∼12%. (e) DF‐STEM image of the region outlined by the white dotted rectangle in (d). (f) BF‐STEM image showing the deformation substructures using diffraction vectors g = 1$\bar{1}$0. (g) DF‐STEM image of the region outlined by the white dotted rectangle in (f). (h) BF‐STEM image showing the deformation substructures at a local strain of ∼45%. (i, j) BF‐STEM images showing dislocation loops, cross kinks/jogs using diffraction vectors g = 110 and g = 211, respectively. (k) Typical IFFT image revealing the Burgers vector a/2 <1$\bar{1}0$> of an edge dislocation (blue line) and the atomic arrangement around another edge dislocation (highlighted in purple). In (k), ‘S’ and ‘F’ indicate the starting and ending points of a Burgers circuit, respectively, whereas the red arrow near ‘S’ and ‘F’ reveals the Burgers vector direction.

Further detailed deformation substructures are analyzed by STEM, as shown in Figure 4d–k. At the early uniform deformation stage (*ε*_loc_ ∼12%), dislocation networks consisting of planar dislocation channels on primary <111>{110} slip systems become predominant (Figure 4d). Within these channels, wavy dislocations with an average spacing of approximately 50 nm, occasionally accompanied by dislocation loops, are arranged in ordered patterns as presented in Figure 4e. Outside the channels, screw dislocations are primarily observed in the form of kink pairs (Figure 4f,g), while straight and long screw dislocations account for only a very small proportion. Dislocation pinning and intersection can also be observed (Figure 4f,g). With increasing local strain to ∼45% at the late uniform deformation stage, the dislocation density in the RHEA increases dramatically. The intense dislocations progressively tangle into high‐density dislocation walls (HDDWs) with spacing in the range of 50–100 nm along intersecting {110} slip planes (Figure 4h). Bright‐field STEM imaging under two‐beam conditions in Figure 4i,j reveals a complex dislocation substructure, featuring frequent cross‐kinks and interactions between dislocations, which evolve into dense tangles, networks, and debris (including jogs, dipoles, and loops). In a representative atomic‐resolution IFFT image in Figure 4k, an edge dislocation with the Burgers vector *a*/2 <1$\bar{1}0$> can be identified. Moreover, the inserted enlarged image, highlighted in purple, further reveals the atomic arrangement around an edge dislocation.

For comparison, the deformation microstructures of the base alloy are shown by EBSD IPF and KAM maps in Figure S10. The results suggest that the severely deformed grains are elongated along the tension direction (Figure S10a–c) and the GNDs are also inhomogeneously distributed in the base alloy (Figure S10d–f). Electron channeling contrast (ECC) images show that planar slip bands dominate the early deformation stage with *ε*_loc_ of ∼10% (Figure S10g). At the medium uniform deformation stage with *ε*_loc_ of ∼30% (Figure S10h), distinct and regularly arranged planar slips can be observed in grain interiors. During the necking stages (*ε*_loc_ ∼70%, Figure S10i), shear bands become prevalent with grains severely elongated. To further clarify the deformation mechanisms, STEM analysis of the deformed substructures in the base alloy is presented in Figure S11. At the early plastic deformation stage (*ε*_loc_ ∼7%), planar slip is prevalent along the <111>{110} slip system. The dislocation channel networks are built up of edge arrays and straight screws. Almost no additional dislocations are observed outside these channels (Figure S11a–c). With the increase of local strain to ∼52%, the long and straight screw dislocations glide far away from the channel, facilitating intersections that form dislocation networks, accompanied by sporadic pinning points and debris. Notably, kink pairs or cross‐kinks are only infrequently observed. (Figure S11d–f). This contrast in deformation homogeneity is therefore manifested in the fracture morphology, i.e., while both alloys are fractured by microvoid coalescence (see Figure S12), the average dimple size of the RHEA (∼8.35 µm) is ∼60% smaller than that of the base alloy (∼20.6 µm).

The deformation microstructures in the vicinity of fracture surfaces after tensile loading at elevated temperatures were also analyzed by EBSD and STEM methods. According to the EBSD analysis (Figure S13), geometrically necessary dislocations of high number densities were developed throughout the deformed grains after tensile deformation at 773–973 K, whereas local dynamic recrystallization (LDRX) can be observed around the original grain boundaries after loading at temperatures above 1073 K, which correlates to the softening phenomenon shown in Figure S8a. Further detailed STEM analysis in Figure S14 reveals a transition in dislocation morphology with deformation temperature. More specifically, a high number density of dislocation debris and loops was observed in the 773 K deformed grains. At 973 K, well‐defined dislocation networks are formed. At 1073 K, LDRX causes the formation of fine sub‐grains (1–2 µm) with a virtually dislocation‐free interior. At 1173 K, dislocations rearrange into a honeycomb‐like structure at some subgrain boundaries, and dislocation tangles can also be observed in some regions. With increasing temperature to 1273 K, short and straight screw dislocations become dominant, and the total number density of dislocations decreases with temperature.

## 3 Discussion

The present lightweight RHEA is characterized by extraordinary room temperature tensile ductility (∼47.8%) at yield strength exceeding 1 GPa, as can also be underlined by its unprecedentedly high product of specific yield strength and tensile elongation compared to that of the previous RHEAs (Figure 3). The RHEA also exhibits superior specific tensile yield strength from room temperature to 1273 K compared to that of the established superalloys and emerging multi‐principal‐element alloys (Figure S8). The significant enhancement in the mechanical properties of the present RHEA can be primarily attributed to the presence of dispersed CGONs revealed above. The large negative mixing enthalpies of Al‐Zr (−44 kJ/mol) and Al‐Ti (−30 kJ/mol) pairs indicate strong preferential attraction among Al, Zr, and Ti in the Nb‐Zr‐Ti‐Ta‐Al system (Table S1). This can drive the rearrangement of Zr, Ti, and Al to form ordered nanodomains under annealing at appropriate temperatures. Further, the difference in atomic mobility underlies the formation of a compositional gradient in the ordered domains. More specifically, the elements with relatively higher diffusivity (e.g., Ti, Zr and Al) tend to migrate into the core, while the other elements with lower diffusivity (e.g., Nb and Ta, D ≈ 10^−18^ m^2^·s^−1^ at 1273 K) can be kinetically hindered, leading to the chemical gradient. The high melting point implies intrinsically high activation energies for diffusion of the refractory elements in the RHEA [36], whereas the severe lattice distortion can create a rugged energy landscape that further elevates the diffusion barrier. Critically, the resultant CGONs, in which the compositions of the shell region are similar to that of the adjacent BCC matrix (Figure 1d), eliminate sharp chemical interfaces, thereby significantly reducing the coherency strain and interfacial energy (γ_eff_ ≈ 0.025 J·m^−2^) [37, 38]. This lowers the nucleation barrier and enables the precipitation of fairly fine CGONs (2.84 ± 0.38 nm) upon short‐term annealing (Figure 2e). Further, the minimal interfacial energy is conducive to the exceptional short‐term thermal stability, allowing the CGONs to retain their small size (< 5 nm) and uniform distribution during tensile deformation at elevated temperatures (Figure S15), consistent with the sluggish coarsening kinetics predicted by the Lifshitz‑Slyozov‑Wagner theory given the ultra‑low interfacial energy of the CGONs and the slow diffusion of refractory elements [39, 40].

The gigapascal room temperature yield strength of the present fairly ductile RHEA originates from multiple strengthening mechanisms. According to quantitative analysis (see details in Note S1), the strengthening from the massive solid solution, the grain‐boundary strengthening, and the strengthening from dispersed CGONs are estimated to be ∼731, ∼75, and ∼252 MPa, respectively. The strengthening from the massive solid solution, which constitutes the largest contribution, is primarily induced by the lattice distortion and modulus mismatch within the multi‐principal element matrix [41]. Notably, the dispersed CGONs in the matrix provide the second‐largest contribution to the yield strength. Also, the exceptional thermal stability of the CGONs ensures the retention of the potent strengthening effect at elevated temperatures.

The extraordinary ductility of the present RHEA at room temperature is primarily attributed to its prominent capacities for dislocation multiplication and storage. First, the coherent interface with minimal lattice misfit (0.018%) between the CGONs and the matrix relaxes long‐range stress fields and provides transitional pathways for dislocation motion, which significantly facilitates dislocation nucleation and glide at the onset of plastic flow (Figure 4d). Second, the chemical composition of the shell in the CGON is similar to that of the adjacent disordered matrix, and it varies gradually from the shell to the core, exhibiting a relatively low Peierls stress across the CGONs and matrix, facilitating screw dislocation nucleation [42]. More specifically, as dislocations move toward the core region of the CGON, the increasing Zr and Ti concentrations at the core impose progressive lattice friction, and hence, dislocation cores can vary from compact to extended. This transition can lower the energy of screw dislocation cores, promoting dislocation cross‐slip and storage activation [43] (Figure 4e). Third, the CGONs promote kink nucleation and enhance the mobility of screw dislocations by creating gradient compositional fluctuations that reduce the energy barrier of kink formation [44, 45] (Figure 4g; Figure S16). These facilitated kinks can develop on multiple glide planes, and their intersection during glide far away from the dislocation channels leads to the formation of stable cross‐kinks (Figure 4j). These cross‐kinks perform a triple function: (i) mitigate local stress concentrations in planar arrays; (ii) promote massive dislocation multiplication via double cross‐slip and the activation of new Frank‐Read sources; (iii) provide effective obstacles (jogs, dipoles, and loops) [42, 46] that facilitate the formation of dislocation tangles and networks (Figure 4h–j). These mechanisms can largely promote dislocation multiplication to enhance the strain hardening rate and ductility [47].

## 4 Conclusion

In summary, we demonstrated a strategy to develop extraordinarily ductile high‐strength lightweight RHEAs by triggering dispersed coherent CGONs with sizes of several nanometers in the disordered multicomponent matrix. In the CGONs, the chemical composition of the shell is close to that of the matrix, and there is a gradual chemical transition from the shell toward the core. The accordingly designed prototype RHEA features a low mass density (∼6.48 g·cm^−3^), extraordinary ductility (∼21.5% uniform elongation and ∼47.8% total elongation), and high strength (exceeding 1.0 GPa) at room temperature, as well as the exceptional intrinsic high‐temperature mechanical performance. The unique architecture of CGONs ensures high interfacial coherency, minimizes strain energy, enhances thermodynamic stability, and facilitates efficient stress transfer. This architecture also endows the CGONs with robust stability up to 1273 K. The coarsening‑kinetics modeling also suggests that significant degradation is not expected under prolonged intermediate‑temperature service. Upon plastic deformation, the CGONs act as potent gradient‐modulated obstacles to dislocation motion, promoting dislocation nucleation, multiplication, and storage, leading to a sustained high strain hardening rate and exceptional tensile ductility at gigapascal‐level strength. The design concept of CGONs can be extended to other multicomponent systems, opening a new avenue for designing next‐generation high‐performance structural materials for extreme environments.

## 5 Materials and Methods

### 5.1 Thermodynamic Calculations

The compositional design of the present RHEA was guided by systematic thermodynamic calculations using the Pandat 2022 software and the PanNi2022 database. The principal elements Nb, Ti, and Zr were selected to form a stable BCC solid‐solution matrix, with Ti (28.0 at.%) promoting dissolution of other elements due to its low valence‐electron concentration, and Zr (29.5 at.%) and Nb (32.0 at.%) set to near‐equiatomic ratios to maximize solid‐solution strengthening via lattice distortion. Al and Ta were introduced to promote the formation of chemically gradient ordered nanodomains (CGONs). Al exhibits large negative mixing enthalpies with Zr and Ti, providing a strong driving force for ordering, while its low atomic mass reduces overall density, and its smaller atomic size contributes to lattice distortion in the bulk alloy. Ta, being a refractory element with a high melting point, improves high‐temperature stability and provides additional strengthening through modulus mismatch. However, excessive Al or Ta may lead to brittle intermetallic phases or increase density beyond the lightweight target; therefore, their contents must be synergistically optimized. To achieve this, we performed a two‐step CALPHAD screening. First, fixing Al at 8 at.%, we varied Ta from 0 to 5 at.% (Figure S1a) and found that Ta above 2.5 at.% introduces unwanted secondary phases above 500 K, thus limiting Ta to ≤ 2.5 at.%. Second, fixing Ta at 2.5 at.%, we varied Al from 0 to 12 at.% (Figure S1b) and observed that Al below 7 at.% gives a narrow BCC window and weak ordering, while Al above 9 at.% promotes brittle intermetallics; the optimal Al content is 8 at.%, providing the widest single‐BCC temperature range and sufficient driving force for CGON formation. Based on these calculations, the nominal composition Nb_32_Zr_29.5_Ti_28_Ta_2.5_Al_8_ (at.%) was selected for the present RHEA.

### 5.2 Material Preparation

The bulk RHEA ingot with nominal composition Nb_32_Zr_29.5_Ti_28_Ta_2.5_Al_8_ (at.%) was synthesized by arc‐melting a mixture of the constituent elements with purity higher than 99.99 wt.% under a Ti‐gettered argon atmosphere. To minimize the volatilization loss of Al, the elemental Al was placed at the bottom and covered by the heavier elements (Ta, Nb, Zr, Ti); the melting was performed in 5–6 cycles with moderate current and limited melting time (< 2 min per cycle). The exact bulk chemical composition of the as‐cast alloy was analyzed by inductively coupled plasma mass spectrometry (ICP‐MS), and the results are shown in Table S2. The exact chemical compositions of the RHEA are Nb 31.81, Zr 29.65, Ti 28.14, Ta 2.45, and Al 7.83 (at.%), close to the nominal values.

To further guide the thermomechanical processing, we calculated the thermodynamic phase diagram of the Nb_32_Zr_29.5_Ti_28_Ta_2.5_Al_8_ (at.%) RHEA. As shown in Figure S1c, there is a wide BCC phase region at temperatures above 500–1650 K, suggesting that the present RHEA can obtain a BCC matrix with the tuning of ordering features by appropriate thermal treatments. Based on the above considerations, we selected the temperature of 1123 K for annealing. Alloy plates with a thickness of 10 mm were cut from the as‐cast ingot and then cold‐rolled with a thickness reduction ratio of ∼90%. Plate specimens were later wire‐cut from the cold‐rolled sheets and then annealed at 1123 K for 5 min in vacuum, followed by water quenching.

### 5.3 Materials Characterization

Phase identification of the various samples was performed by X‐ray diffraction (XRD) analysis using a Rigaku Smartlab D/max 2550 VB X‐ray diffractometer with Cu‐kα_1_ (λ = 1.54056 Å) operated at 40 kV and 30 mA. The measurement was carried out at a scanning speed of 4°/min. Back‐scattered electron imaging (BSEI) and electron channeling contrast imaging (ECCI) were coupled with electron backscattered diffraction (EBSD) to reveal the microstructures using a TESCAN CLARA field emission gun scanning electron microscope (SEM) with a high‐resolution camera and a TSL OIM data collection software. More detailed substructures were characterized by an FEI‐TALOS transmission electron microscope (TEM) equipped with energy dispersive spectroscopy (EDS) at an acceleration voltage of 200 kV. Strain++ software was used to characterize the lattice strain distribution based on corresponding high‐resolution (HR) TEM images. Atom probe tomography (APT) measurement was conducted using a local CAMECA LEAP 5000XR electrode atom probe in laser‐assisted mode. The APT data were reconstructed and analyzed using AP Suite Software 6.1 (CAMECA Instrument). Neutron diffraction analysis was performed on a General‐Purpose Power Diffractometer (GPPD) with a 360° rotation stage at China Spallation Neutron Source (CSNS).

The surfaces of all samples used in XRD, BSEI, and ECCI measurements were successively ground and polished with silicon carbide paper, diamond suspension, and colloidal silica to obtain a mirror‐like surface. For TEM sample preparation, foils with a thickness less than 80 µm were prepared by mechanical grinding, and they were made into discs with a 3‐mm diameter, followed by electro‐polishing at −30°C using a twin‐jet polisher with a solution consisting of 6 vol% perchloric acid, 35 vol% butanol, and 59 vol% water at a voltage of 30 V. The APT and the high‐temperature tensile deformation samples were prepared with the focused‐ion beam (FIB) lift‐out technique on an FEI Dual‐beam microscope.

The tensile samples are flat dog‐bone shaped with a total length of 21 mm (gauge length of 8 mm, gauge width of 2.5 mm) and a thickness of 1.5 mm. Before tensile testing, the surfaces of the flat specimens were ground using 400‐, 800‐, 1200‐, 3000‐, and 5000‐grit SiC paper. Uniaxial tensile tests were performed at a constant engineering strain rate of 1 × 10^−3^ s^−1^ at room temperature, and a digital image correlation (DIC) system was used to measure the local strain evolution during room‐temperature tensile testing. High temperature tensile tests were performed using a universal testing machine (INSTRON 3382) equipped with a resistive heating furnace. Tensile specimens were heated to target temperatures (773, 873, 973, 1073, 1173, and 1273 K, respectively) at a constant heating rate of 2 K s^−1^, held for 5 min to ensure thermal uniformity, and then strained at a constant rate of 1 × 10^−3^ s^−1^, followed by furnace cooling. To guarantee the reliability and repeatability of the tensile results, at least three samples were tested for each condition. To retain and reveal the deformation microstructures in the vicinity of fracture surfaces after tensile loading at elevated temperatures, high‐temperature tests were also conducted in a Gleeble‐3500 thermomechanical simulator under a vacuum of 10^−3^ mbar, with identical heating and straining procedures, followed by quenching in high‐pressure helium gas immediately after fracture.

## Author Contributions

**Wei Zhang**: investigation, methodology, data curation, formal analysis, writing – original draft. **Dingshun Yan**: methodology, validation, resources, software. **Yong Zhang**: methodology, validation, visualization. **Zhiming Li**: conceptualization, methodology, investigation, formal analysis, supervision, resources, writing – review and editing, funding acquisition, project administration.

## Funding

The work is financially supported by the Scientific Research Innovation Capability Support Project for Young Faculty (Grant No: ZYGXQNJSKYCXNLZCXM‐M27), the Major Fundamental Research Program of Hunan Province (Grant No: 2024JC0003), and the Science and Technology Innovation Program of Hunan Province (Grant No: 2023RC1013).

## Conflicts of Interest

The authors declare no conflicts of interest.

## Supporting information




**Supporting File**: advs76407‐sup‐0001‐SuppMat.docx.

## Data Availability

All data needed to evaluate the conclusions are present in the paper and Supporting Information. Additional data can be obtained from the corresponding author under reasonable conditions.
