# Supplementary material for: Chemically Gradient Ordered Nanodomains Enable Large Tensile Ductility in Gigapascal Lightweight Refractory High‐Entropy Alloys
Source: Adv Sci (Weinh). 2026 Jul 6:e76407. Online ahead of print. doi: 10.1002/advs.76407 (PMC13335762; doi:10.1002/advs.76407)
Supplement: Supplementary file 1 — Supporting File: advs76407‐sup‐0001‐SuppMat.docx. [file ADVS-9999-e76407-s001.docx]

Supporting Information for

**Chemically** **gradient ordered nanodomains** **enable large tensile ductility in** **gigapascal** **lightweight** **refractory** **high-entropy alloys**

Wei Zhang^1^, Dingshun Yan^1^, Yong Zhang^1^, Zhiming Li^1,2*^

W. Zhang, D. Yan, Y. Zhang, Z. Li

1. School of Materials Science and Engineering, Central South University, Changsha 410083, China

Z. Li

2. State Key Laboratory of Powder Metallurgy, Central South University, Changsha 410083, China

***^*^***Correspondence to: [lizhiming@csu.edu.cn](mailto:lizhiming@csu.edu.cn) (Z. Li)

**This file includes:**

**Notes S1**

**Figures S1 to S16**

**Tables S1 to S3**

**References**

**Note S1: Evaluation of strengthening mechanisms**

The yield strength of the RHEA is quantitatively evaluated ($\sigma_{y}^{cal}$) based on the cumulative effects from various strengthening mechanisms, i.e.,

$\sigma_{y}^{cal}={\Delta\sigma}_{is}+{\Delta\sigma}_{gb}+{\Delta\sigma}_{ds}$ (1)

where ${\Delta\sigma}_{is}$, ${\Delta\sigma}_{gb}$ and ${\Delta\sigma}_{ds}$represent intrinsic solid-solution strengthening, grain-boundary strengthening and dispersion strengthening by the CGONs, respectively. It is worth noting that intrinsic solid solution strengthening comprises two components: intrinsic lattice friction (${\Delta(\sigma_{0.2})}_{mix}$) and solid solution strengthening (${\Delta\sigma}_{ss}$).

${\Delta(\sigma_{0.2})}_{mix}=\sum_{i=1}^{n} c_{i}({\sigma_{y})}_{i}$, $c_{i}$ and $({\sigma_{y})}_{i}$ are the atomic ratio and yield strength of each constituent element. Thus, $\Delta{(\sigma_{0.2})}_{mix}$ is evaluated to be 154 MPa.

A Labusch-type solution strengthening model applicable to RHEA is used to estimate ${\Delta\sigma}_{ss}$[1]:

${\Delta\sigma}_{ss}=\frac{G}{45}{(\sum_{i} \varepsilon_{i}^{2}c_{i})}^{2/3}$ (2)

where $G=\sum_{i=1}^{N} x_{i}G_{i}$ is the shear modulus of solvent matrix, 𝑥_𝑖_ and 𝐺_𝑖_ are the atomic friction and the shear modulus of each constituent element *i*. *c_i_* are the shear modulus and solute concentration of *i* the element, respectively, the *ε_i_* is the misfit parameter, which includes atomic misfit *ε_ri_* and modulus misfit *ε_Gi_*. The relationship of *ε_i_* with *ε_ri_* and *ε_Gi_* is defined as:

$\varepsilon_{i}={(\varepsilon_{ri}^{2}+a^{2}\varepsilon_{Gi}^{2})}^{1/2}$ (3)

Here, *a* is a constant (2-16) and depends on the type of mobile dislocations (screw or edge)[2]. In the RHEA, the dislocations are a mixture of different types, and so it is taken as 9. The atomic radius, shear modulus and yield strength of pure metals were given in Table S3, as well as *ε_ri_* and *ε_Gi_*. The calculated strength from the solid solution strengthening of the RHEA is 576 MPa. Thus, the intrinsic solid-solution strengthening is 731MPa.

The effect of grain boundary strengthening on yield stress has a close relationship with the average grain size. The grain boundary strengthening $\Delta$σ*_gb_* of the RHEA can be calculated via the following formula, i.e.,

$\Delta\sigma_{gb}=k_{hp}d^{-1/2}$ (4)

Herein, Hall-Petch coefficient (*k_hp_*) is 244 MPa·μm^1/2^[3], average grain size (*d*) is~10.32 µm, as measured experimentally. Thus, values of strength increment from grain boundary strengthening ($\Delta$σ*_gb_*) are calculated to be ~75 MPa.

CGON strengthening originates from the interactions between dislocations and CGONs, three contributing factors are mainly considered in the calculation of the effect of CGONs sheared by dislocations. They are CGONs matrix coherency ($\Delta$σ*_cs_*), modulus mismatch ($\Delta$σ*_ms_*) and atomic ordering ($\Delta$σ*_os_*), which could be expressed as:

${\Delta\sigma}_{ds}={\Delta\sigma}_{cs}+{\Delta\sigma}_{ms}+{\Delta\sigma}_{os}$ (5)

${\Delta\sigma}_{cs}=M\alpha_{\varepsilon}{(G\varepsilon_{a})}^{\frac{3}{2}}{(\frac{rf_{P}}{0.5Gb})}^{\frac{1}{2}}$ (6)

${\Delta\sigma}_{cs}=0.0055M{\Delta G}^{\frac{3}{2}}{(\frac{2f_{P}}{G})}^{\frac{1}{2}}{(\frac{r}{b})}^{\frac{3m}{2}-1}$ (7)

${\Delta\sigma}_{os}=0.81M\frac{\gamma_{APB}}{2b}{(\frac{3\pi f_{B2}}{8})}^{\frac{1}{2}}$ (8)

where *M* = 2.73 is the Taylor factor; *α_ε_*= 2.6 (constant) for a BCC structure; $\varepsilon_{a}=\frac{2}{3}(\frac{\Delta a}{a})$ is the constrained lattice parameter mismatch with $\Delta$*a* being the lattice parameter difference between the CGONs and the matrix (0.09Å); *a* being the lattice parameter of the matrix (3.383Å); *r* (2.86 nm) is the average radius; $f_{P}$ (9.6%) is the volume fraction of the CGONs; *b* is the magnitude of the Burgers vector; γ*_APB_*=0.111mJ/m^2^ is the average antiphase boundary energy of CGON[4]; and *m* is a constant taken to be 0.85[5]. It is calculated that the $\Delta$σ*_ds_* is estimated as 252 MPa.

Based on the above discussion and calculation, the theoretically evaluated $\sigma_{0.2}^{cal}$ = 1058 MPa agrees well with the measured $\sigma_{0.2}^{exp}$ = 1032 MPa.

**Figures S1 to S16**


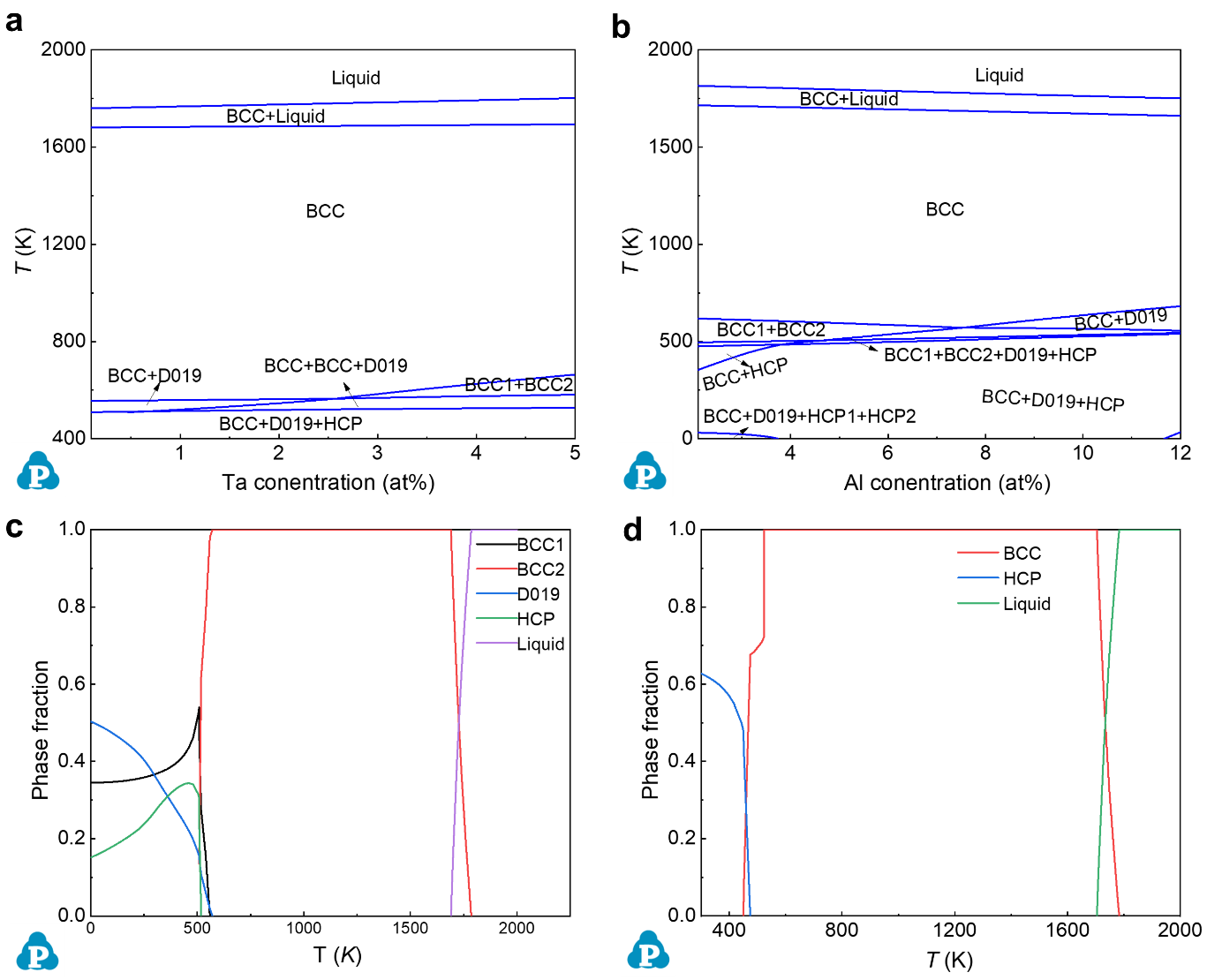


**Figure S1** Calculated equilibrium phase diagrams for designing the refractory high-entropy alloys (RHEAs). (a) (Nb_32_Zr_29.5_Ti_28_Al_8_) _(100-_*_x_*_)_ Ta*_x_* (0 ≤ *x* ≤5) system. (b) (Nb_32_Zr_29.5_Ti_28_Ta_2.5_) _(100-_*_x_*_)_ Al*_x_* (0 ≤ *x* ≤12) system. (c) The Nb_32_Zr_29.5_Ti_28_Ta_2.5_Al_8_ (at. %) RHEA. (d) The reference Nb_34.0_Zr_33.0_Ti_33.0_ (at. %) base alloy.


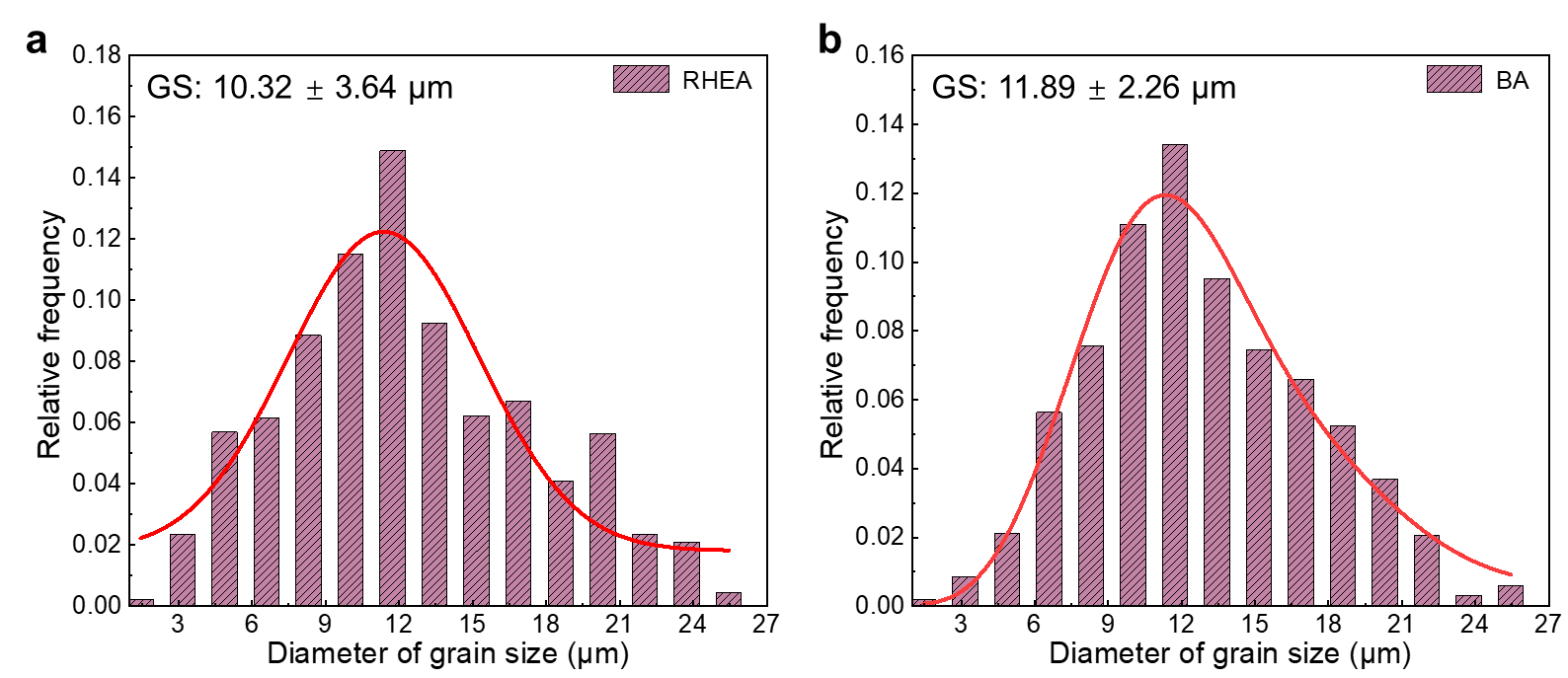
**Figure S2** Grain size distribution histograms of the RHEA and the reference alloy. (a) RHEA (Nb_32_Zr_29.5_Ti_28_Ta_2.5_Al_8_) with average grain size of 10.32 ± 3.64 µm. (b) Reference alloy (BA) with average grain size of 11.89 ± 2.26 µm. The grain sizes were measured from EBSD data.


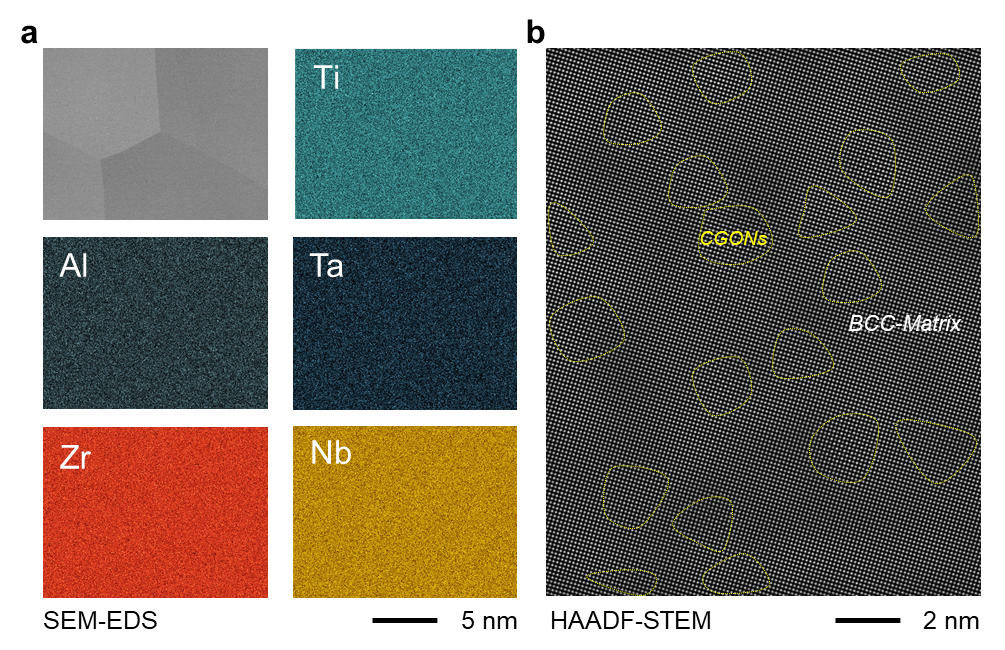


**Figure S3** Microscale elemental distribution and atomic-scale structure of the RHEA. (a) BSE image and the corresponding EDS maps showing that all elements (i.e., Nb, Zr, Ti, Ta and Al) are uniformly distributed at the micro-scale. (b) HAADF-STEM images the [001] zone axis showing the atomic-scale structure of the ordered CGONs.


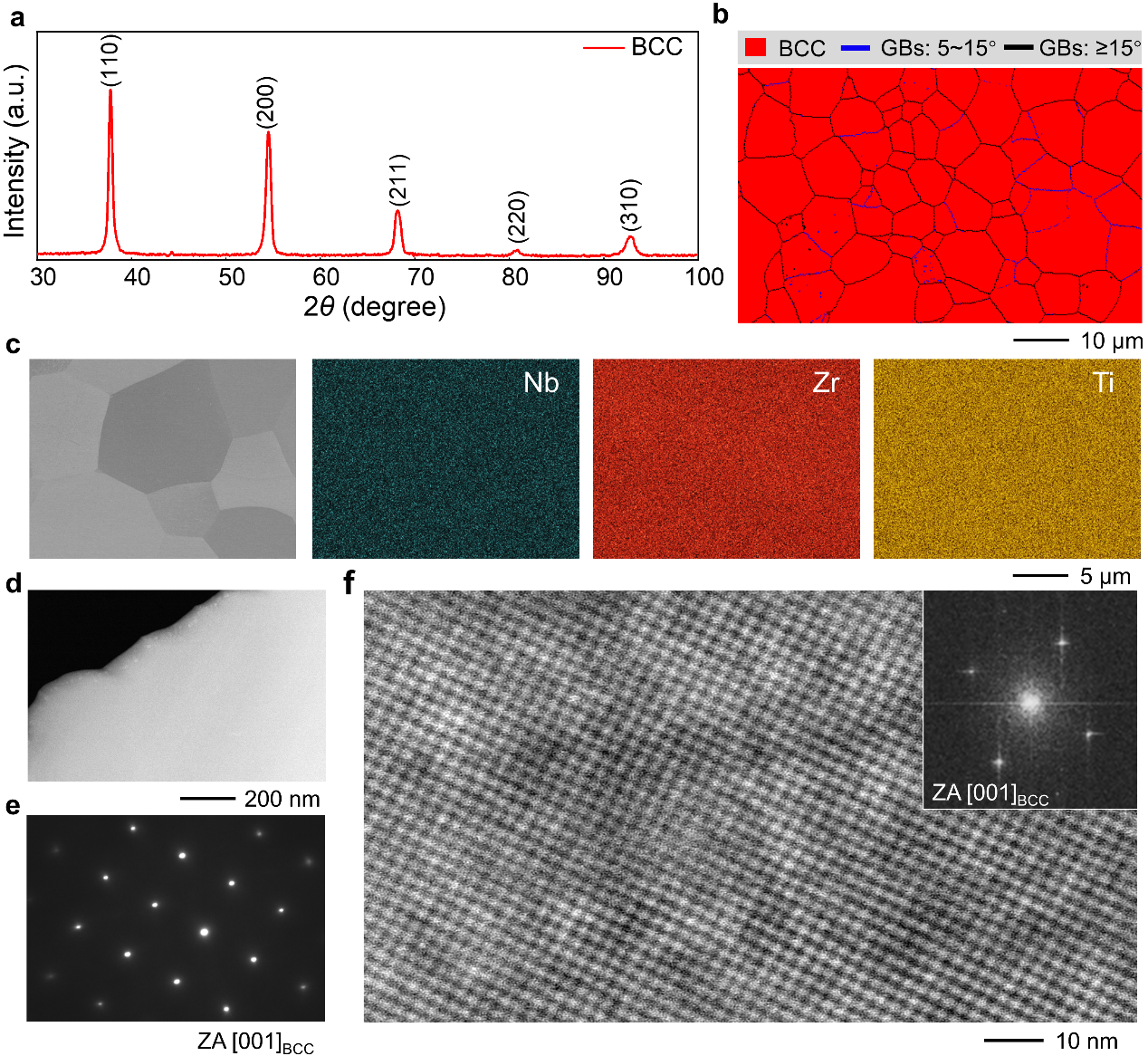


**Figure S4** Elemental distribution and structural characterization of the reference Nb_34.0_Zr_33.0_Ti_33.0_ base alloy. (a, b) XRD pattern and EBSD phase map showing a BCC structure. The term “a.u.” refers to “arbitrary unit”. Source data are provided as a Source Data file. (c) BSE image and the corresponding EDS maps showing the uniformly distributed elements (i.e., Nb, Zr and Ti) at micro-scale. (d) Bright-field STEM image showing the nano-scale morphology. (e) SAED pattern taken along the [001] zone axis. (f) HAADF-STEM image with the [001] zone axis and the corresponding FFT pattern (inset).

**Figure S5** Size distribution histogram of CGONs measured from APT reconstruction.

**
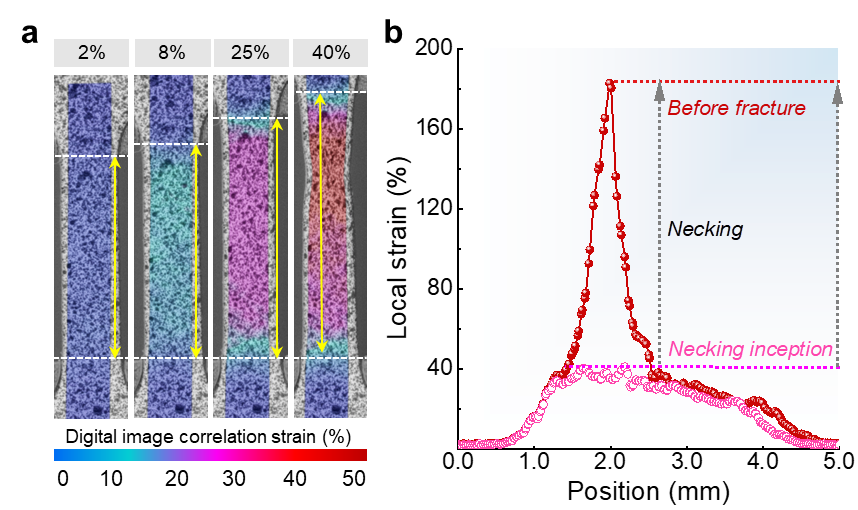
**

**Figure S6** (a) Two-dimensional strain distribution maps at different loading stages measured by the digital image correlation (DIC) method. (b) Local strain profiles for the RHEA at the necking inception point (haloed circles) and one frame before fracture (solid circles), obtained from in situ digital image-correlation tensile tests.

**
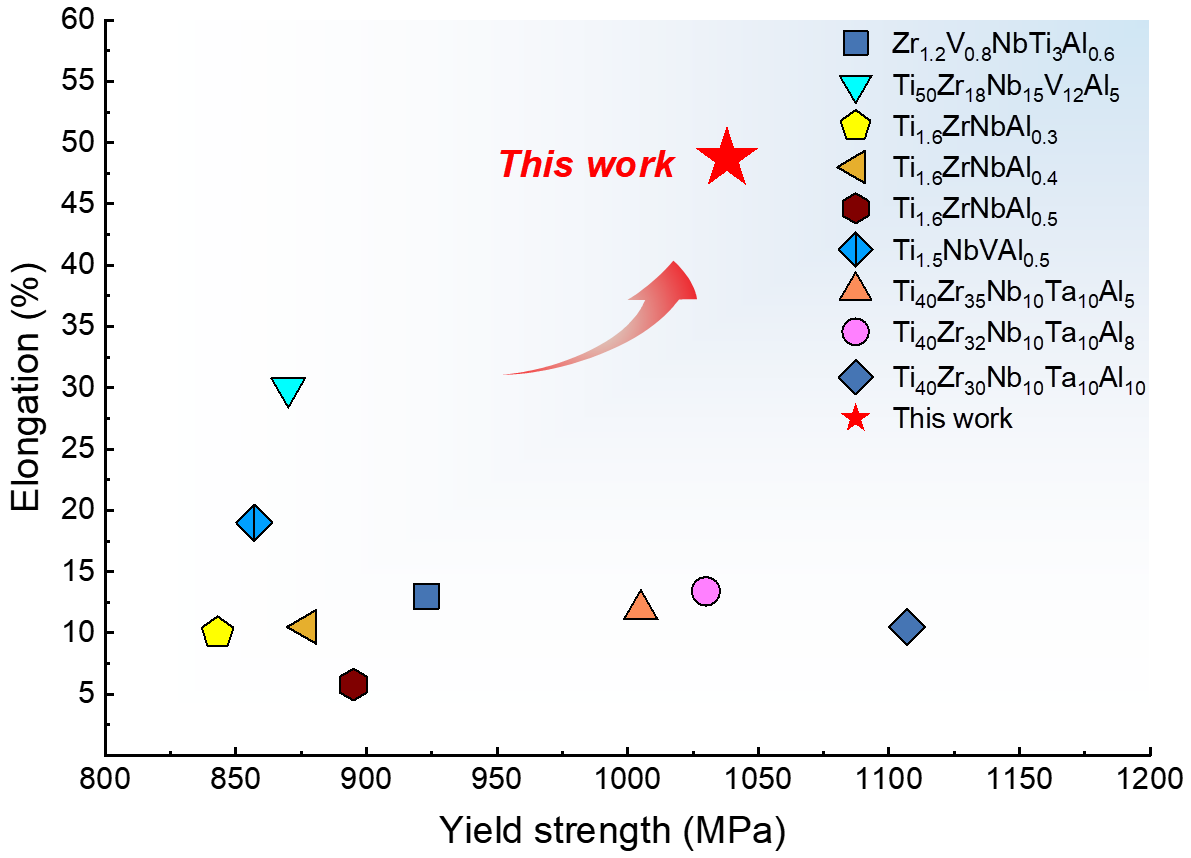
**

**Figure S7** Yield strength versus tensile elongation of the RHEA compared to other alloys reinforced with conventional B2 precipitates[6-11].

**
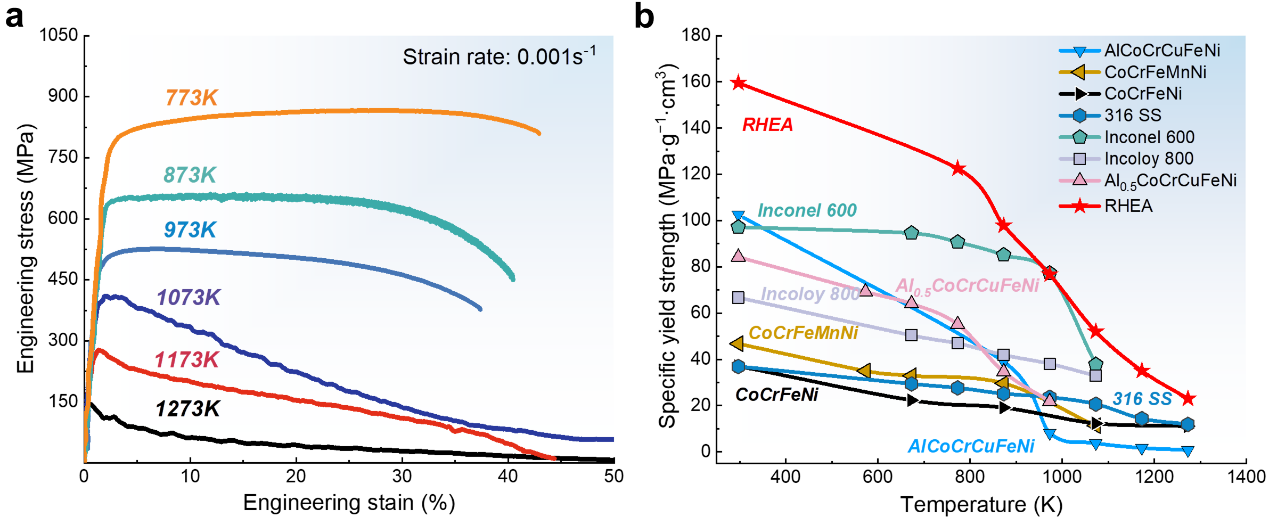
**

**Figure S8** Mechanical properties of the present lightweight RHEA at elevated temperatures. (a) Typical tensile engineering stress-strains curves obtained at temperatures from 773 K to 1273 K at a strain rate of 1$\text{×}$10^-3^ s^-1^. (b) Plots of specific tensile yield strength with respect to temperature for the present RHEA, previous superalloys and emerging multi-principal-element alloys[12-18]. Note that some other intrinsic refractory multi-principal-element alloys (e.g., WTaMoNb and WTaMoNbV) typically lack measurable tensile ductility but only compressive results could be obtained, and thus they are not included in the present comparisons of tensile properties.


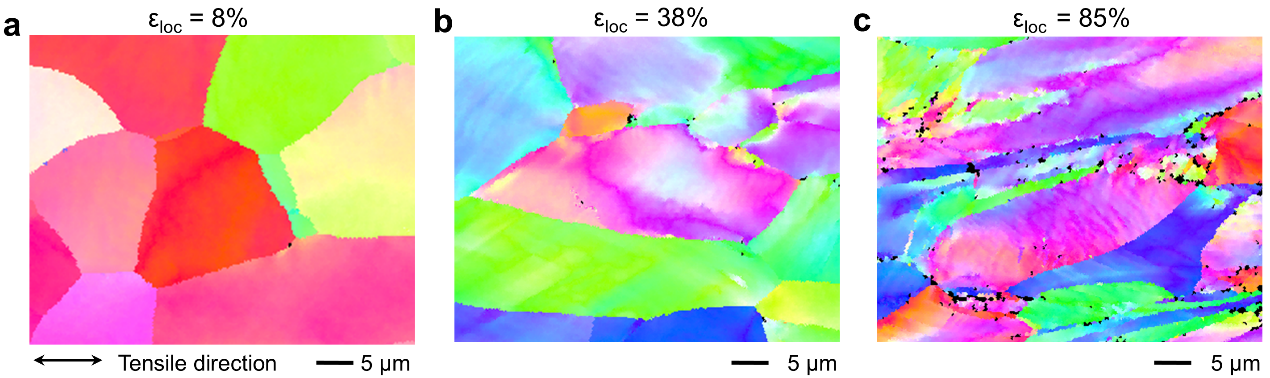


**Figure S9** (a-c) Inverse pole figure (IPF) maps of the RHEA showing microstructural evolution at different true strain levels (*ε*_loc_ ~ 8%, 38%, and 85%).


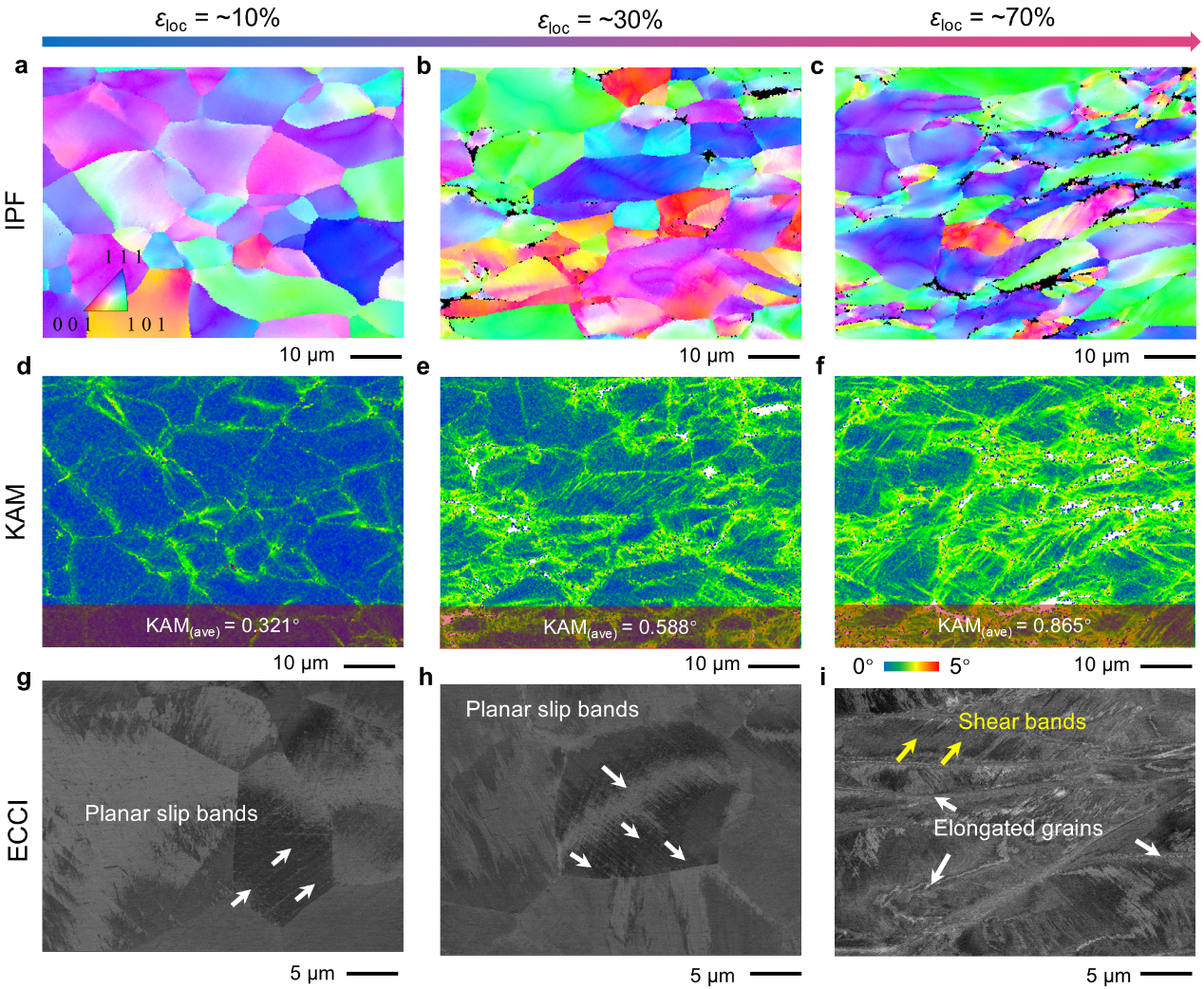


**Figure S10** Microstructural evolution of the base alloy under tensile deformation at true strain (*ε*_loc_) levels of ~10%, ~30%, and ~70%, respectively. (a-c) IPF maps. (d-f) The corresponding kernel average misorientation (KAM) maps. (g-i) Electron channeling contrast (ECC) images revealing the microstructural evolution.


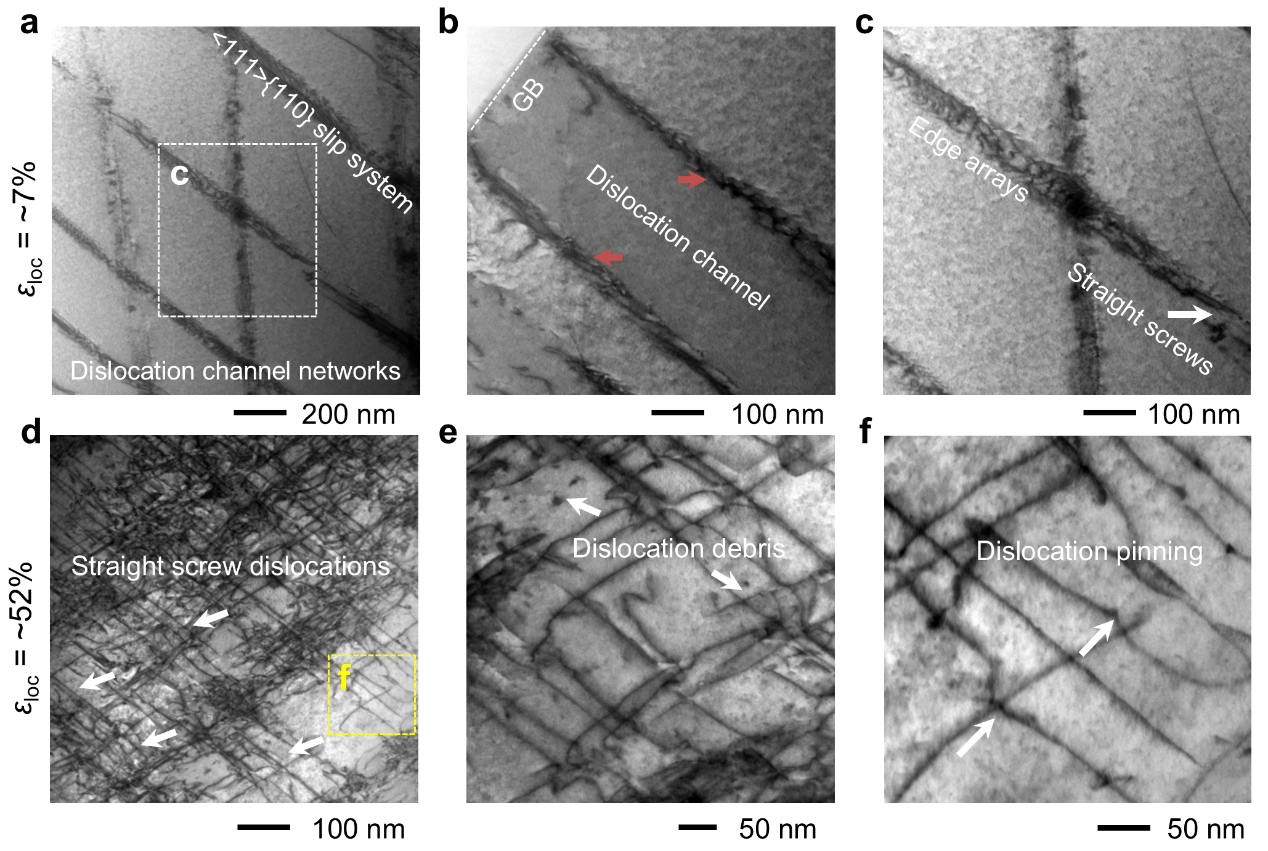


**Figure S11** TEM images showing the deformation microstructures of the base alloy. (a-c) Bright-field STEM images at a local strain (*ε*_loc_) of ~7%. (d-e) Bright-field STEM image at a local strain (*ε*_loc_) of ~52%.


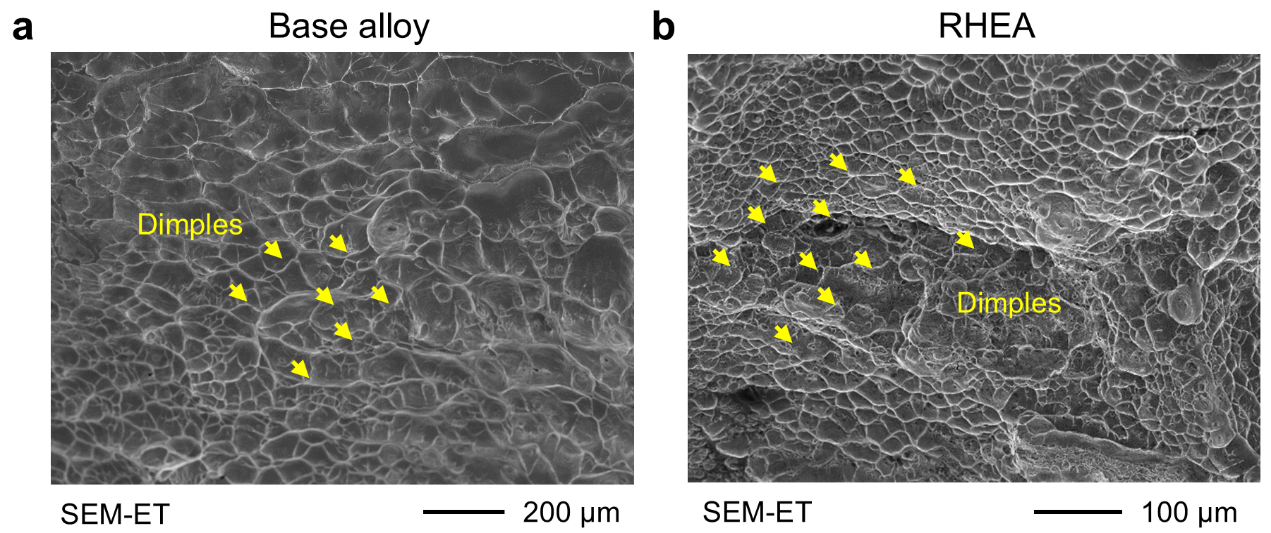


**Figure S12** SEM images of the fracture surfaces after uniaxial tensile testing. (a) Fracture morphology of the base alloy, revealing large and shallow dimples. (b) Fracture morphology of the RHEA, showing smaller and deeper dimples than the base alloy.


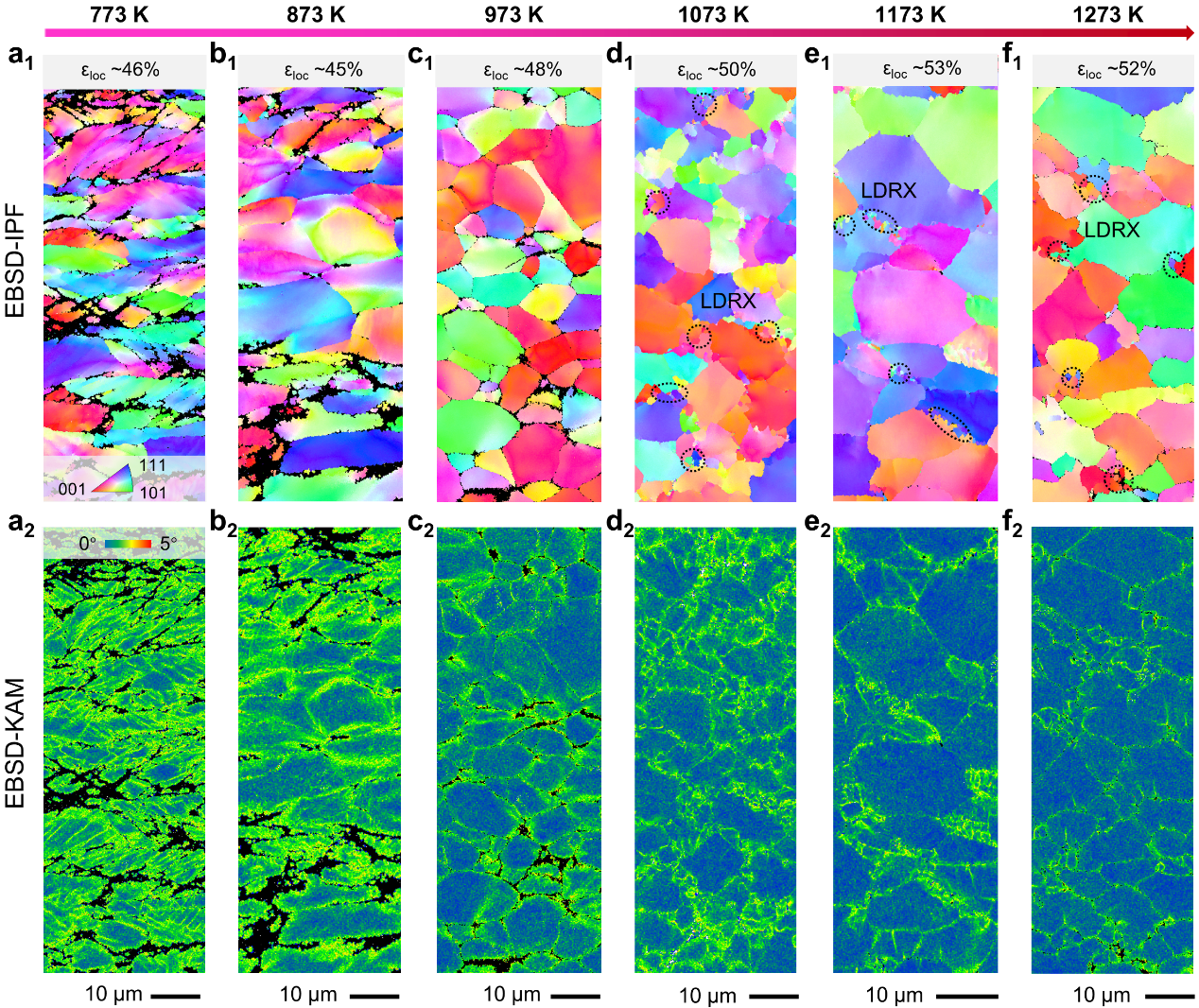


**Figure S13** Microstructural evolution of the RHEA during high-temperature tensile deformation based on EBSD analysis. (a_1_-f_1_) EBSD IPF maps obtained in the sample region near to the fracture surface after tensile testing at temperatures from 773 K to 1273 K. The exact local strain values vary from 45% to 53% in the samples fractured at different temperatures, as marked on the images. (a_2_-f_2_) The corresponding EBSD kernel average misorientation (KAM) maps. Local dynamic recrystallization (LDRX), indicated by black dashed circles in (d_1_-f_1_), occurs preferentially near original grain boundaries, upon tensile deformation at 1073 K and above.

_
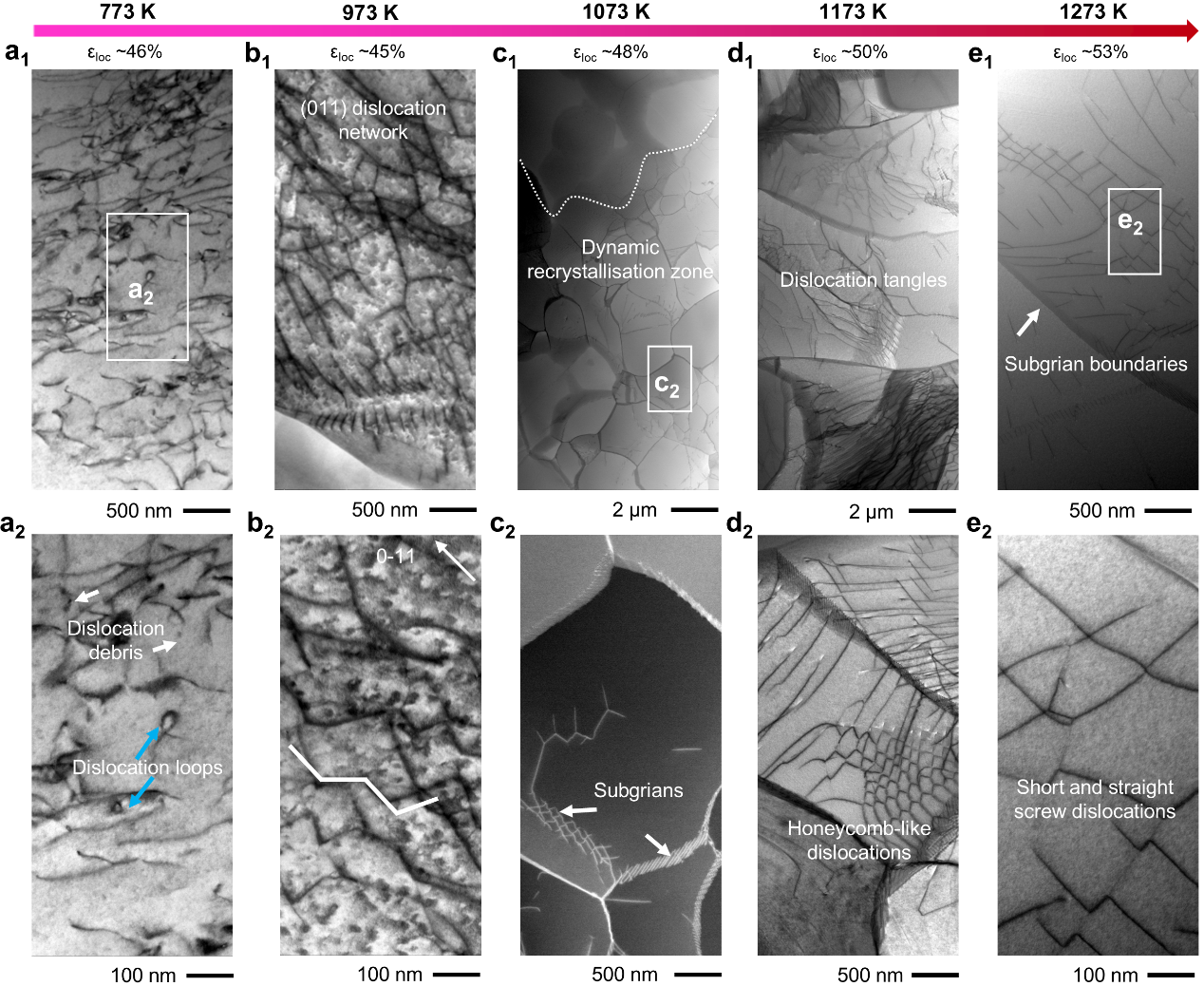
_

**Figure S14** Deformation substructures of the lightweight RHEA at high temperature based on STEM analysis. (a_1-2_) STEM images obtained from the sample deformed at 773 K with a local strain of 46%. (b_1-2_) STEM images for the sample after 973 K deformation at a local strain of 45%. (c_1-2_) STEM images for the sample deformed at 1073 K with a local strain of 48%. (d_1-2_) STEM images obtained from the sample after 1173 K deformation at a local strain of 50%. (e_1-2_) STEM images for the sample deformed at 1273 K with a local strain of 53%. To unambiguously characterize and compare the dislocation behaviors, all STEM images (a_1_-e_1_) were acquired with the electron beam aligned along the [111] zone axis. The dark spots visible in panels (b_1_-b_2_) refer to the local damage resulting from ion-thinning during sample preparation and do not affect the analysis of dislocation configurations.

**
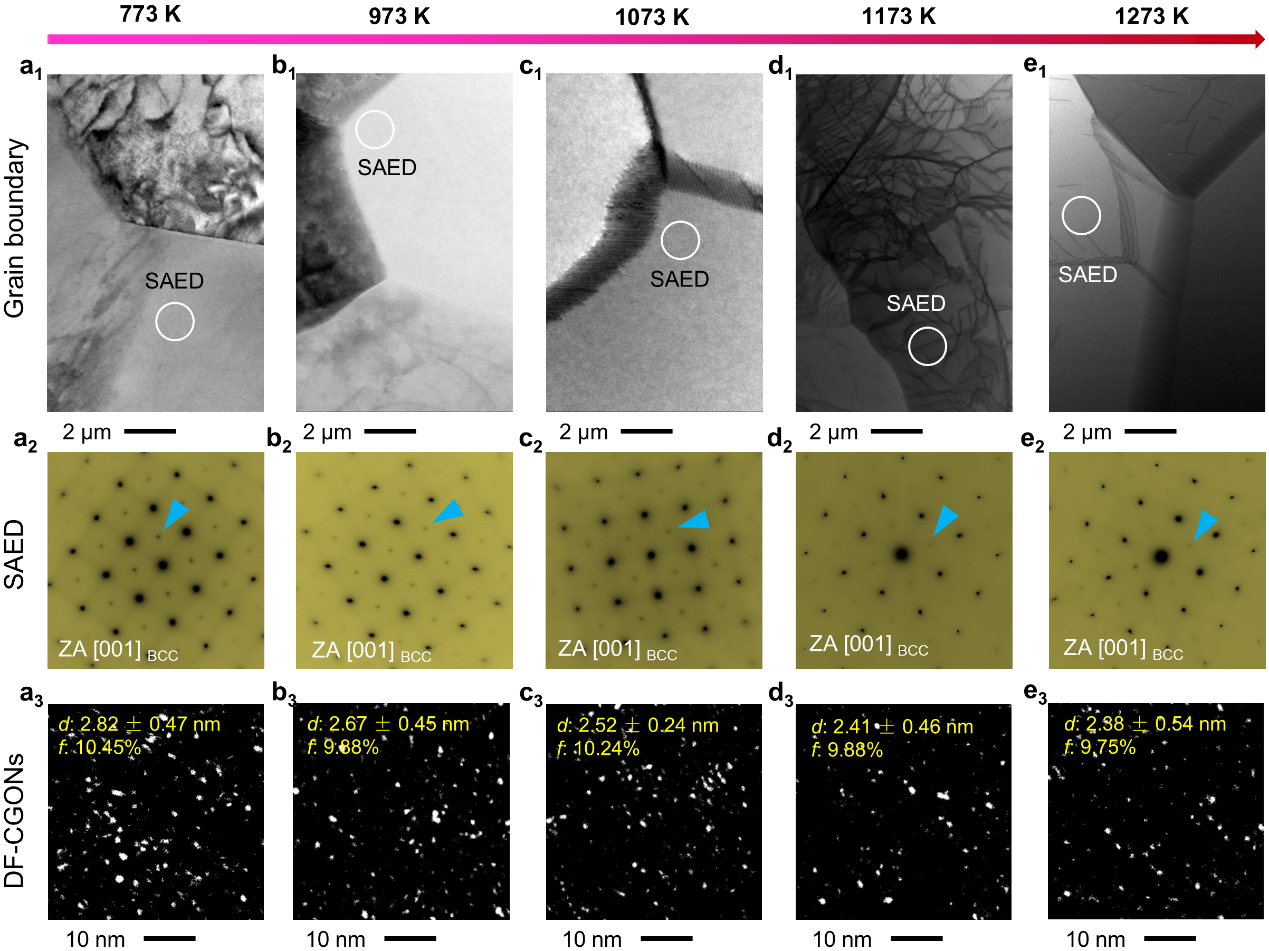
**

**Figure S15** Structure of the CGONs after high temperature tensile tests. (a_1_-e_1_) Morphologies of typical grain boundary regions in the samples after tensile deformation at 773 K, 973 K, 1073 K, 1173 K and 1273 K, respectively. (a_2_-e_2_) SAED patterns corresponding to the sample regions marked in (a_1_-e_1_). (a_3_-e_3_) Dark-field TEM images taken by the selected diffraction spots of CGONs marked in (a_2_-e_2_), respectively. Note: The SAED and dark‑field images clearly demonstrate that the CGONs remain ordered and coherent after deformation.


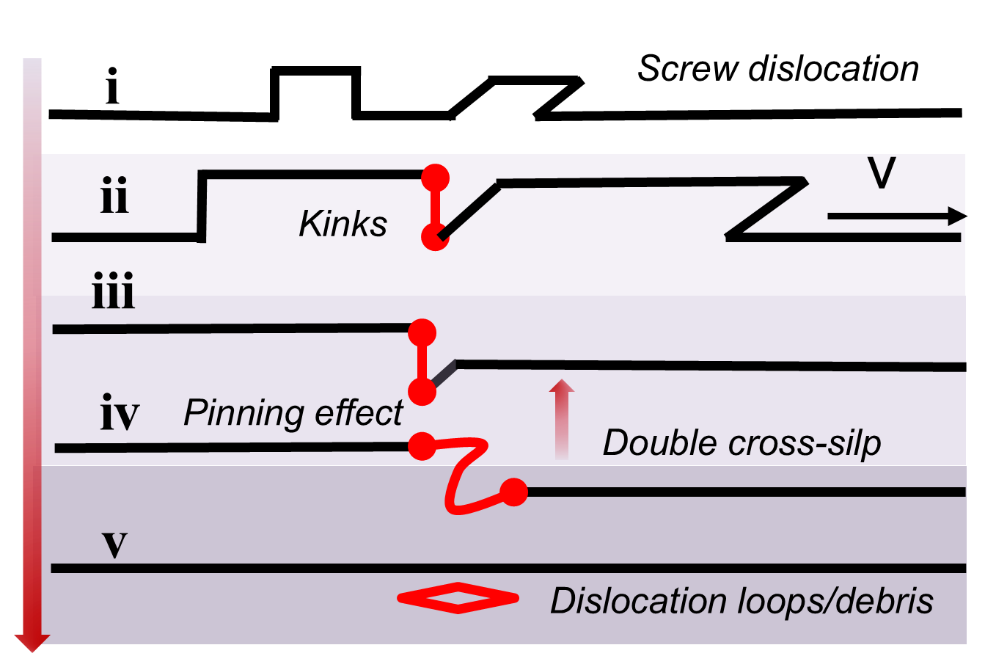


**Figure S16** Schematic diagram of the “kink-pairs” mechanism: (**ⅰ-ⅱ**) Formation of a super jog at the meeting point of two kinks travelling in different planes; (**ⅲ**) Dislocation is pinned by the super jog; (**ⅵ-ⅴ**) The dislocation arm on the right cross-slips moves back to the initial glide plane, resulting in the formation of a dislocation loop which lies on the plane perpendicular to the primary plane.

**Tables S1 to S3**

**Table S1** Mixing enthalpies (*∆H_mix_*) for alloying element pairs in NbZrTiTaAl system[19].

| *∆H_mix_* | Ti | Zr | Ta | Al |
| --- | --- | --- | --- | --- |
| Nb | 2 | 4 | 0 | -18 |
| Zr |  |  | 3 | -44 |
| Ti |  |  | -1 | -30 |
| Ta |  |  |  | -7 |

**Table S2** Nominal and measured chemical compositions (at.%) of the RHEA determined by inductively coupled plasma mass spectrometry (ICP-MS).

| Element (at. %) | Nb | Ti | Zr | Ta | Al |
| --- | --- | --- | --- | --- | --- |
| Nominal composition | 32.00 | 29.50 | 28.00 | 2.50 | 8.00 |
| Actual composition | 31.82 | 28.14 | 29.65 | 2.45 | 7.93 |

**Table S3** Basic date for the elements in the RHEA. The atomic radius, *r*, shear modulus, *G*, and tensile yield stress, *σ_py_*, Lattice distortion, *ε_ri_*, and modulus distortion *ε_Gi_* of pure metals Nb, Ti, Zr, Ta and Al, respectively.

| Properties | Nb | Ti | Zr | Ta | Al |
| --- | --- | --- | --- | --- | --- |
| *r* ($Å$) | 1.429 | 1.462 | 1.603 | 1.460 | 1.432 |
| *G* (GPa) | 38 | 45 | 35 | 69 | 25 |
| $\sigma_{py}$ (MPa) | 105 | 140 | 207 | 705 | 30 |
| *K_hp_* (MPa μm^1/2^) | 340 | 190 | 280 | 760 | 90 |
| *ε_ri_* | -0.054 | -0.022 | 0.139 | 0.002 | -0.011 |
| *ε_Gi_* | -0.085 | 0.192 | -0.220 | 0.073 | -0.190 |

**References**

[1] S.I. Rao*, et al.*, *Acta Materialia* 209 (2021): 116758.

[2] M. Lugovy, V. Slyunyayev,M. Brodnikovskyy, *Progress in Natural Science: Materials International* 31(1) (2021): 95-104.

[3] C. Wang*, et al.*, *Materials Science and Engineering: A* 941 (2025): 148574.

[4] A.K.g. D. banerjee, and T.K. nandy, *Metallurgical and Materials Transactions A* 21 (1990): 627.

[5] N. Yurchenko*, et al.*, *Materials Research Letters* 10(12) (2022): 813-823.

[6] L. Lilensten*, et al.*, *Materials Research Letters* 5(2) (2016): 110-116.

[7] C.-C. Juan*, et al.*, *Materials Letters* 184 (2016): 200-203.

[8] O.N. Senkov,S.L. Semiatin, *Journal of Alloys and Compounds* 649 (2015): 1110-1123.

[9] Y. Wu*, et al.*, *Materials Science and Engineering: A* 724 (2018): 249-259.

[10] W. Lai*, et al.*, *Materials Research Letters* 10(3) (2022): 133-140.

[11] Z. Lei*, et al.*, *Nature* 563(7732) (2018): 546-550.

[12] B. Schuh*, et al.*, *Acta Materialia* 96 (2015): 258-268.

[13] A. Gali,E.P. George, *Intermetallics* 39 (2013): 74-78.

[14] N. Stepanov*, et al.*, *Intermetallics* 59 (2015): 8-17.

[15] G.A. Salishchev*, et al.*, *Journal of Alloys and Compounds* 591 (2014): 11-21.

[16] S. Goyal*, et al.*, *Materials Science and Engineering: A* 696 (2017): 407-415.

[17] L.Y. Xu*, et al.*, *Engineering Failure Analysis* 31 (2013): 375-386.

[18] C.-W. Tsai*, et al.*, *Journal of Alloys and Compounds* 490(1-2) (2010): 160-165.

[19] A.T.a.A. Inoue, *Materials Transactions* 46(12) (2005): 2817-2829.
